# Persistent organic pollutants and endogenous sex-related hormones in Hispanic/Latino adults: The Hispanic Community health study/study of Latinos (HCHS/SOL)

**DOI:** 10.1016/j.envres.2024.120742

**Published:** 2024-12-30

**Authors:** Chibuzor Abasilim, Victoria Persky, Robert M. Sargis, Tessa Day, Konstantina Tsintsifas, Martha Daviglus, Jianwen Cai, Sally Freels, Arielle Grieco, Brandilyn A. Peters, Carmen R. Isasi, Gregory A. Talavera, Bharat Thyagarajan, Mark Davis, Richard Jones, Andreas Sjodin, Mary E. Turyk

**Affiliations:** a Division of Epidemiology and Biostatistics, School of Public Health, University of Illinois Chicago, USA; b Division of Environmental and Occupational Health Sciences, School of Public Health, University of Illinois Chicago, USA; c Division of Endocrinology, Diabetes, and Metabolism, Department of Medicine, University of Illinois Chicago and Medical Service, Jesse Brown VA Medical Center, USA; d Institute for Minority Health Research, University of Illinois Chicago, USA; e Department of Biostatistics, University of North Carolina at Chapel Hill, USA; f Department of Epidemiology and Population Health, Albert Einstein College of Medicine, USA; g Department of Psychology, San Diego State University, USA; h Department of Laboratory Medicine and Pathology, University of Minnesota, USA; i Persistent Pollutants Biomonitoring Laboratory, Centers for Disease Control and Prevention, Atlanta, GA, USA

**Keywords:** Hispanics/latinos, Persistent organic pollutants, Sex hormones, Diabetes, Body mass index, Endocrine disruption

## Abstract

**Background:**

Previous studies have demonstrated associations of persistent organic pollutants (POPs) with sex-related hormones; however, findings were inconsistent. Sex-specific impacts and pathways through which adiposity influences associations are not completely understood. We sought to evaluate sex-specific associations of POPs serum concentration with sex-related hormones and to explore pathways through which adiposity may modify associations.

**Methods:**

We studied 1073 men and 716 postmenopausal women participating in the “Persistent Organic Pollutants, Endogenous Hormones, and Diabetes in Latinos” ancillary study which is a subcohort of the “Hispanic Community Health Study/Study of Latinos.” We use baseline examination data collected from 2008 to 2011 to investigate associations between eight organochlorine pesticides (OCPs), five polychlorinated biphenyls (PCB) groups, sum of polybrominated diphenyl ethers and polybrominated biphenyl 153 on sex hormone binding globulin (SHBG) and various sex-related hormone levels. We examined associations cross-sectionally using linear and logistic regression models adjusted for complex survey design and confounders.

**Results:**

PCBs and select OCPs were associated with increased SHBG in women and decreased estradiol (E2) and/or bioavailable E2 in men. For instance, per quartile increase in serum concentrations of ∑PCBs and oxychlordane were associated with decreased levels of E2 (β = −6.36 pmol/L; 95% CI: 10.7,−2.02 and β = −5.08 pmol/L; 95% CI: 8.11,−2.05) and bioavailable E2 (β = −4.48 pmol/L; 95% CI: 7.22,−1.73 and β = −4.23 pmol/L; 95% CI: 6.17,−2.28), respectively, in men, and increased levels of SHBG (β = 7.25 nmol/L; 95% CI:2.02,12.8 and β = 9.42 nmol/L; 95% CI:4.08,15.0), respectively, in women. p,p’-DDT and β-HCCH, and o,p’-DDT were also associated with decreased testosterone (T) and bioavailable T (ng/dL) levels in men. Adiposity modified associations in men, revealing stronger inverse associations of PCBs, PBDEs, and several OCPs with LH, SHBG, E2, bioavailable E2, T, and the ratios of LH to FSH and E2 to T in those with below median body mass index and waist-to-hip ratio.

**Conclusion:**

Distinct patterns of hormone dysregulation with increasing POPs serum concentration were identified in men and post-menopausal women. In men but less so in postmenopausal women, adiposity modified associations of POPs serum concentration with sex-related hormones.

## 1. Introduction

Exposure to persistent organic pollutants (POPs) is linked to endocrine disruption which in turn have been associated with cancer, reproductive toxicity, and cardiometabolic diseases including diabetes and hypertension (Sharpe and Irvine, 2004; Vested et al., 2014; Wahlang, 2018; Persky et al., 2023; Lee et al., 2006, 2010; Arrebola et al., 2014). POPs are stable and lipophilic chemicals with elimination half-lives ranging from months to years including among others polybrominated diphenyl ethers (PBDEs), polychlorinated biphenyls (PCBs), and organochlorine pesticides (OCPs) many of which are now withdrawn from the market or banned in the United States (U.S) but were widely used in agricultural and industrial sectors (Pestana et al., 2014; Agency for Toxic Substances and Disease Registry ATSDR, 2021a; Agency for Toxic Substances and Disease Registry ATSDR, 2021b; Agency for Toxic Substances and Disease Registry ATSDR, 2021c; Agency for Toxic Substances and Disease Registry ATSDR, 2008). PCBs are organochlorine compounds that were extensively used in the manufacture of electrical equipment including use as lubricants and coolants (Agency for Toxic Substances and Disease Registry ATSDR, 2021a). PBDEs and polybrominated biphenyl (PBB) are organobromine compounds, previously included in building materials, electronics, textiles, plastics and various products due to their flame-retardant properties (Agency for Toxic Substances and Disease Registry ATSDR, 2021b; Agency for Toxic Substances and Disease Registry ATSDR, 2021c). Dichlorodiphenyltrichloroethane [DDT], predominantly used as an insecticide for malaria vector control along with its isomers (2-(4-chlorophenyl)-2-(2-chlorophenyl)-1,1,1-trichloroethane [o, p’-DDT], and 1,1,1-trichloro-2,2-diphenylethane [p,p’-DDT], and metabolite 1,1-dichloro-2,2-bis(p-chlorophenyl) ethylene [p,p’-DDE] have been extensively measured in people, with studies showing differences in levels across racial and ethnic groups within the U.S. (Agency for Toxic Substances and Disease Registry (ATSDR), 2008; Sjödin et al., 2014) Hexachlorobenzene [HCB], used as a fungicide and in synthetic rubber production, is also a by-product of municipal waste incineration. Beta-hexachlorocyclohexane [β-HCCH], a by-product of lindane production, was previously used in cotton farming, and for treating lice and scabies (Agency for Toxic Substances and Disease Registry (ATSDR), 2008). Mirex, previously used to control fire ants and marketed using the trade name dechlorane, was also used as a flame retardant in various products (Agency for Toxic Substances and Disease Registry (ATSDR), 2008). Oxychlordane and *trans*-nonachlor, both chlordane-related compounds, were widely used for seed treatment, as insecticides on crops, and for termite control (Agency for Toxic Substances and Disease Registry (ATSDR), 2008; Sjödin et al., 2014). Ongoing exposure to POPs occurs through ingestion of contaminated food, in particular animal fat, fish, dairy products, and eggs, and contaminated dust, and to a lesser extent inhalation of contaminated air (Pestana et al., 2014; Agency for Toxic Substances and Disease Registry ATSDR, 2021a).

The endocrine system through sex-steroid pathways, is important for maintaining homeostasis and regulating metabolic processes that impact bone health, reproduction, cardiovascular, and immune function (Hiller-Sturmhöfel and Bartke, 1998). The hypothalamicpituitary-gonadal (HPG) axis regulates levels of circulating sex steroids (estrogens and androgens) secreted from the gonads through an integrated feedback system that includes the pituitary hormones luteinizing hormone (LH) and follicle-stimulate hormone (FSH) (Hiller-Sturmhöfel and Bartke, 1998). Dehydroepiandrosterone (DHEA) produced in the adrenal glands is a precursor of sex steroids (Hiller-Sturmhöfel and Bartke, 1998). Additionally, sex hormone binding globulin (SHBG) regulates bioavailability of testosterone (T) and estradiol (E2) by binding to these hormones in the blood stream, thereby inhibiting their receptor activation and subsequent effects (Hiller-Sturmhöfel and Bartke, 1998). Although previous studies have evaluated associations of PBDEs (Johnson et al., 2013; Turyk et al., 2008; Gao et al., 2016; Makey et al., 2016; Eskenazi et al., 2017), OCPs (Freire et al., 2014; Ferguson et al., 2012; Blanco-Muñoz et al., 2012; Persky et al., 2001; Langer et al., 2010; Martin et al., 2002; Ayotte et al., 2001; Bornman et al., 2018; Dalvie et al., 2004; Giwercman et al., 2006; Madrigal et al., 2021) and PCBs (Goncharov et al., 2009; Persky et al., 2011, 2012; Van et al., 2018; Turyk et al., 2006; Sun et al., 2017; Shi et al., 2020; Lambertino et al., 2021; Pan et al., 2019) with sex-related hormones, findings have been inconsistent highlighting the need for further research.

Previous research evaluating the relationship of POPs with sex-related hormones have several limitations that the present study aims to address. Some studies relied on house dust measurements of PBDEs for exposure assessment while others assessing PCBs and OCPs did not measure a comprehensive range of analytes within the target population. Many studies reported insufficient sample size, which may limit statistical power to detect significant associations impacting adequate subgroup stratification which is crucial for elucidating pathways involved in the relationship of POPs with sex hormones. Most previous studies also focused on men and boys with few including post-menopausal women, a group with elevated risk for cardiovascular disease due to changes in endocrine profile with menopause. Moreover, a majority of prior studies did not account for participants’ use of medications that can substantially alter sex hormone levels and potentially confound the relationship of POPs with sex-related hormones.

In addition, the specific biological mechanisms underlying differences in the association of POPs with sex-related hormones across levels of adiposity remains underexplored. Given the interplay between adipose tissues and the liver in regulating endocrine function, energy homeostasis, and metabolism of xenobiotics and lipids, we hypothesize that adiposity may modify sex-specific associations of POPs with sex-related hormones, which is biologically plausible, of clinical and epidemiologic interest, and supported by previous research (Law et al., 2014; Koceva et al., 2024; Pott et al., 2021; Arsenescu et al., 2008; Chevrier et al., 2000; Dirinck et al., 2011; Wolff et al., 2007; Aaseth et al., 2022; Rhyu and Yu, 2021; Kumar et al., 2014).

We sought to evaluate sex-specific associations of POPs serum concentration with sex-related hormones and indicators of hypothalamic-pituitary-gonadal (HPG) axis regulation in Hispanic/Latino adults 45–74 years of age. We also assessed effect modification on associations of POPs with sex-related hormones through measures of adiposity (body mass index (BMI) and waist-hip-ratio). We used data from an ancillary study nested within the Hispanic Community Health Study/Study of Latinos (HCHS/SOL) which additionally measured POPs and endogenous thyroid and sex-related hormones (Persky et al., 2023). Previous studies in this area do not reflect the heterogeneity within the Hispanic/Latino population, a largely immigrant group demonstrating higher body burden of pesticides and disparities in cardiometabolic disease risk (Sjödin et al., 2014; Haw et al., 2021; Elfassy et al., 2020; Daviglus et al., 2012). Including persons of diverse Hispanic/Latino backgrounds captures variations in socio-cultural factors, potential exposure sources, and body burden of pesticides within this population (Elfassy et al., 2020; Daviglus et al., 2012).

## 2. Methods

### 2.1. Study population

Previous literature provides a comprehensive overview of the current ancillary study “Persistent Organic Pollutants, Endogenous Hormones, and Diabetes in Latinos” including details of the database and study methods (Persky et al., 2023; Abasilim et al., 2023). Briefly, baseline examination (V1) was carried out between 2008 and 2011. HCHS/SOL obtained institutional review board approval from the coordinating center, central laboratory and each field center. As previously described, a case-cohort design was utilized, selecting one individual per household from participants who returned for the first follow-up reexamination (V2) after 6 years. Selection was stratified by sex and glucose levels at V1 and individuals with diabetes and pre-diabetes at V2 were oversampled (Persky et al., 2023). The design of the present ancillary study excluded individuals who did not provide HCHS/SOL consent, were diabetic, had no baseline measurements of lipids or were younger than 45 or older than 74 years. This decision was based on a primary interest to elucidate the interplay of POPs, endogenous hormones and inflammation on the development of diabetes in a subgroup who may be disproportionately impacted by this disease (Persky et al., 2023). The definition of menopause status utilized in the present analyses has been previously described (Persky et al., 2023). Of the 2343 ancillary study participants, the current analysis excluded peri/pre-menopausal women (n = 363), and both men and women using hormone-related medications (n = 191) as previously described (Abasilim et al., 2023). Our final study population included 716 post-menopausal women and 1073 men 45–74 years of age (Table S1).

### 2.2. Sex-related hormones

The outcome of interest was endogenous sex-related hormones. We examined eight sex-related hormones – LH [mIU/mL], FSH [mIU/mL], DHEAS [umol/L], SHBG [nmol/L], estradiol (E2) [pmol/L]; and T [ng/dL] measured only in men. Bioavailable T (ng/dL) and bioavailable E2 (pmol/L) concentrations in men were calculated using Mazer’s method, based on each individual’s concentrations of SHBG (nmol/L), T (ng/dL), E2 (pmol/L) and a constant albumin value of 4.3 g/dL (Mazer, 2009). Stored fasting serum samples were analyzed for hormones using FDA-approved assays. Assays were independently validated with external proficiency testing materials. We have previously described details of the laboratory assay, limit of detection (LOD) and coefficient of variation for individual sex-related hormones (Persky et al., 2023). Hormone assays for this study were performed at the Advanced Research & Diagnostics Laboratory at the University of Minnesota. We analyzed E2 as above or below the LOD in postmenopausal women because 71% of women had E2 measurements below the LOD. In addition, we did not measure T in women due to the limited volume of stored biospecimen. We examined ratio of LH to FSH in both men and postmenopausal women as an indicator of the HPG axis regulation, and ratios of bioavailable E2 to bioavailable T and E2 to T in men only as measures indicating aromatase enzyme activity as previously described (Sollberger and Ehlert, 2016; Banaszewska et al., 2003; He et al., 2007). In men, the reference range of hormones were LH:1.7–8.6 mlU/mL, FSH:1.5–12.4 mlU/mL, E2: 41.4–159 pmol/L, DHEAS:0.44–13.4 μmol/L, SHBG:10–80 nmol/L and T:193–836 ng/dL. In postmenopausal women, the reference range were LH:7.7–58.5 mlU/mL, FSH:25.8–134.8 mlU/mL, E2:<18.4–505 pmol/L, SHBG:20–130 nmol/L, and DHEAS:0.26–11.0 μmol/L.

### 2.3. Persistent organic pollutants

Measurements of POPs were performed at the Centers for Disease Control and Prevention (CDC) laboratories on 1.0 g (quartile range 0.92–1.13) fasting serum samples collected at HCHS/SOL V1. The CDC laboratory does not consider coded specimens to constitute engagement in human subject research. The serum samples were extracted by an automated liquid-liquid extraction procedure (Gilson, Middleton, Wisconsin, USA) and co-extracted materials were removed on an automated solid phase extraction system (Biotage, Upsala, Sweden) using a two-layer cleanup cartridge containing silica and acid silica. Samples were analyzed using a gas chromatography isotope dilution high-resolution mass spectrometry with laboratory quality assurance practices regularly monitored. Each batch of samples constituted 24 unknowns, 3 method blanks, and 3 quality control samples. Our study measured forty-three analytes including twenty-four PCB congeners/co-elutants (IUPAC nos. 105, 114, 118, 138–158, 156, 153, 156, 157, 167, 170, 178, 180, 183, 187, 189, 194, 196–203, 199, 206, 209, 28, 66, 74, and 99), ten PBDEs congeners (IUPAC nos. 100, 153, 154, 17, 183, 209, 28, 47, 85, and 99), PBB 153 and eight OCPs (p,p’-DDT, HCB, β-HCCH, *trans*-nonachlor, p,p’-DDE, mirex, oxychlordane, and o,p’-DDT). The CDC does not report a single LOD value for each analyte because the LOD varies for each sample per analyte and differs based on the sample volume. For each analyte, we imputed values below the LOD reported for each sample as the sample LOD divided by the square root of two (Sjödin et al., 2004). In addition, the overall percent of samples with non-reportable results was 1% for the 43 analytes, with a maximum of 3.5% for individual analytes.

As previously described, we examined POPs serum concentrations as lipid standardized POPs (ng/g lipid) calculated as (wet-weight POPs/total serum lipids (mg/dL) * 102.6) (Phillips et al., 1989). Total serum lipids (mg/dL) were assessed as “(2.27 * total cholesterol (mg/dL) + triglycerides (mg/dL) + 62.31)” (Phillips et al., 1989). Our study examined individual OCPs and PBB-153, as well as PBDEs and PCBs summed based on structure, biological and pharmacokinetic function (Table S2). (Warner et al., 2012) Given that 45%, 54% and 93% of concentrations were below the LOD for mirex, p,p’-DDT, and o,p’-DDT respectively (Table S1), we categorized o,p’-DDT as detected vs non-detected, and mirex and p,p’-DDT as non-detected, below or equal to median of detected values, and above median of detected values. Median detected values were 2.7 ng/g lipid in men and 2.35 ng/g lipid in postmenopausal women for mirex, and 4.04 ng/g lipid in men and 8.59 ng/g lipid in postmenopausal women for p,p’-DDT. We also conducted sensitivity analyses to account for the impact of only including PCB and PBDE congeners with detection frequencies above 60% and 75% (Appendix S1). Associations of summed PCB and PBDE groups with sex-related hormones were similar in both primary and sensitivity models (not shown in tables).

### 2.4. Covariates

We used a directed acyclic graph (DAG) and evidence from prior studies (Ferguson et al., 2012; Persky et al., 2001; Madrigal et al., 2021) to consider covariates for inclusion in multivariable models (Fig. S1). We included participants age (continuous in years), HCHS/SOL study recruitment sites (Miami, FL; Bronx, NY; San Diego, CA; and Chicago, IL), educational attainment (categorized as having below high school diploma, having high school diploma or General Education Development Diploma (GED), and having above high school diploma or GED) and Hispanic/Latino background (Cuban, Dominican, South American, Central American, more than one background, Mexican, or Puerto Rican). We also included physical activity levels categorized as low, moderate, and high (Hoos et al., 2012–07), alcohol use (categorized as no current alcohol use, low level use, and high level use), diet quality based on the Alternative Healthy Eating Index score from 2010 (Chiuve et al., 2012) and smoking status (categorized as never, former, or current smoker). We included waist-to-hip ratio calculated as waist circumference (cm) divided by hip circumference (cm) and BMI calculated as weight (kg) divided by height (m^2^). We also included estimates of glomerular filtration rate (eGFR) based on serum cystatin C, serum creatinine, gender, age and race (Inker et al., 2012); a measure of acculturation using the Multi-Ethnic Study of Atherosclerosis (MESA) score based on nativity, years of residence in the USA, and language spoken at home (Kandula et al., 2008); and number of live births among postmenopausal women based on maternal recall. We controlled for the number of live births in postmenopausal women to account for hormonal changes in women attributed to pregnancy and the variability in body burden of POPs following child birth and breast feeding (Solomon and Weiss, 2002). Prediabetes status at V1 was identified as a potential collider (Fig. S1) and was not controlled for in models (Banack and Kaufman, 2015).

### 2.5. Statistical analyses

The present analysis accounted for the complex survey design of the HCHS/SOL and subsequent selection into the ancillary study. We utilized analytical methods that included the clustered sampling design, stratification and sampling weights. We evaluated missing data on hormones, POPs, and covariates (Table S1) and utilized multiple imputation methodology [STATA MICE procedure (version 17.0)] to address missingness as previously described in other ancillary study analysis (Abasilim et al., 2023). Sex stratification was done *a priori* for the present analysis using the STATA subpopulation command. We assessed survey weighted means or geometric means (95% confidence intervals) and frequencies (percentages) for continuous and categorical variables, respectively. Likewise, differences were evaluated using T-tests and χ^2^ test for continuous and categorical variables, respectively.

We used multivariable linear regression to evaluate sex-specific cross-sectional associations of POPs serum concentration with sex-related hormones. We also modeled associations of POPs with low E2 (above or below the LOD) using multivariable logistic regression in postmenopausal women. We examined serum POPs concentrations (ng/g lipid) in quartiles to address non-linearity except for mirex, p,p’-DDT and o,p’-DDT categorized as previously described. When quartiles of POPs had p for trend ≤0.05, we examined the size and direction of estimates across higher quartiles compared to the lowest quartile. Because our analysis suggested that the effect across quartiles were linear, we present our findings primarily as per quartile increase in POPs serum concentration. We also assessed linear regression model assumptions, addressing violations of the normality assumption, by natural log (Ln) transformation of SHBG in postmenopausal women as well as SHBG, LH, and FSH in men. To maintain consistency, estimates for associations of POPs when hormones are Ln transformed were presented as transformed back calculated values with the following formula [100 (*e^βPOPS^* −1)%] (Barrera-Gómez and Basagaña, 2015).

Models adjusted for age, BMI, waist-to-hip ratio, acculturation score MESA, HCHS/SOL study sites, Hispanic/Latino background, educational attainment, eGFR, cigarette use, alcohol consumption, physical activity levels, AHEI-2010 and number of live births in postmenopausal women only. We examined BMI and waist-to-hip ratio for evidence of multi-collinearity and independence (spearman’s correlation coefficient for BMI and waist-to-hip ratio was 0.5 in men and 0.15 in postmenopausal women) before inclusion in the final model as previously described in other analysis of the ancillary study (Persky et al., 2023). Variance of inflation values ranged between 1.1 and 1.8 for BMI and/or waist-to-hip ratio supporting null evidence of multi-collinearity in models including both BMI and waist-to-hip ratio.

We also tested effect modification of adiposity (BMI and waist-to-hip ratio) on associations of POPs concentrations with sex-related hormones by including a product term (continuous measures of adiposity*POPs concentrations) to models and controlling for covariates in final models. When product terms suggested effect modification based on p-value ≤0.05, we stratified models by median BMI (28.1 kg/m^2^ in men and 29.2 kg/m^2^ in postmenopausal women) and waist-hip-ratio (0.96 in men and 0.91 in postmenopausal women). We did not stratify across BMI categories using the World Health Organization criteria due to smaller cell sizes within the underweight and normal weight categories. Stratification at median BMI values in exploratory analysis showed that sex-specific stratification at these values produced more robust categories for subgroup comparisons and were overall consistent with obesity vs other category.

We also conducted sensitivity analyses to assess robustness of findings. First, we conducted exploratory mediation analysis through adiposity by comparing estimates of association between POPs and sex-related hormones in sensitivity models excluding both measures of adiposity - BMI and waist-to-hip ratio to primary models which included adiposity measures and did not find evidence supporting mediation. Using multivariable logistic regression models, we also explored associations of POPs with sex hormones levels outside the reference range. In men, we evaluated associations of POPs with high LH, FSH, E2 and low T while in postmenopausal women, associations with low SHBG and DHEAS were evaluated. All statistical analyses were carried out using STATA (version 17.0, StataCorp, College Station, TX).

## 3. Results

### 3.1. Descriptive analyses

The present study comprised 716 postmenopausal women and 1073 men aged 45–74 years. Compared to men, postmenopausal women had higher geometric mean concentrations of OCPs and PCBs except for the summed concentration of PCB congeners that are phenobarbital-type inducers (∑PHB-PCB). Men, however had higher geometric mean concentrations of brominated flame retardants – ∑PBDEs and PBB 153 congener (Table 1). Compared to men, postmenopausal women were older (59.3 and 55.5 years), less active and had a higher proportion of obese individuals and those who never smoked or consumed alcohol (Table 1). Mean levels of sex-related hormones by median POPs concentrations (ng/g lipid) and characteristics of study participants are presented in Tables S3–S6. In postmenopausal women, LH, SHBG, and DHEAS levels differed by select POPs (Table S3), whereas in men, FSH, SHBG, E2, bioavailable E2, DHEAS, T, and bioavailable T levels differed by select POPs (Table S4). Overall, sex-related hormone levels differed across several characteristics in both men and postmenopausal women including age, Hispanic/Latino background, smoking status, eGFR concentration, alcohol consumption, BMI, HCHS/SOL study recruitment sites and number of prior live births in women only (Tables S5 and S6).

### 3.2. Multivariable analyses

#### 3.2.1. Association of POPs serum concentration with sex-related hormones in postmenopausal women

**Fig. 1.**
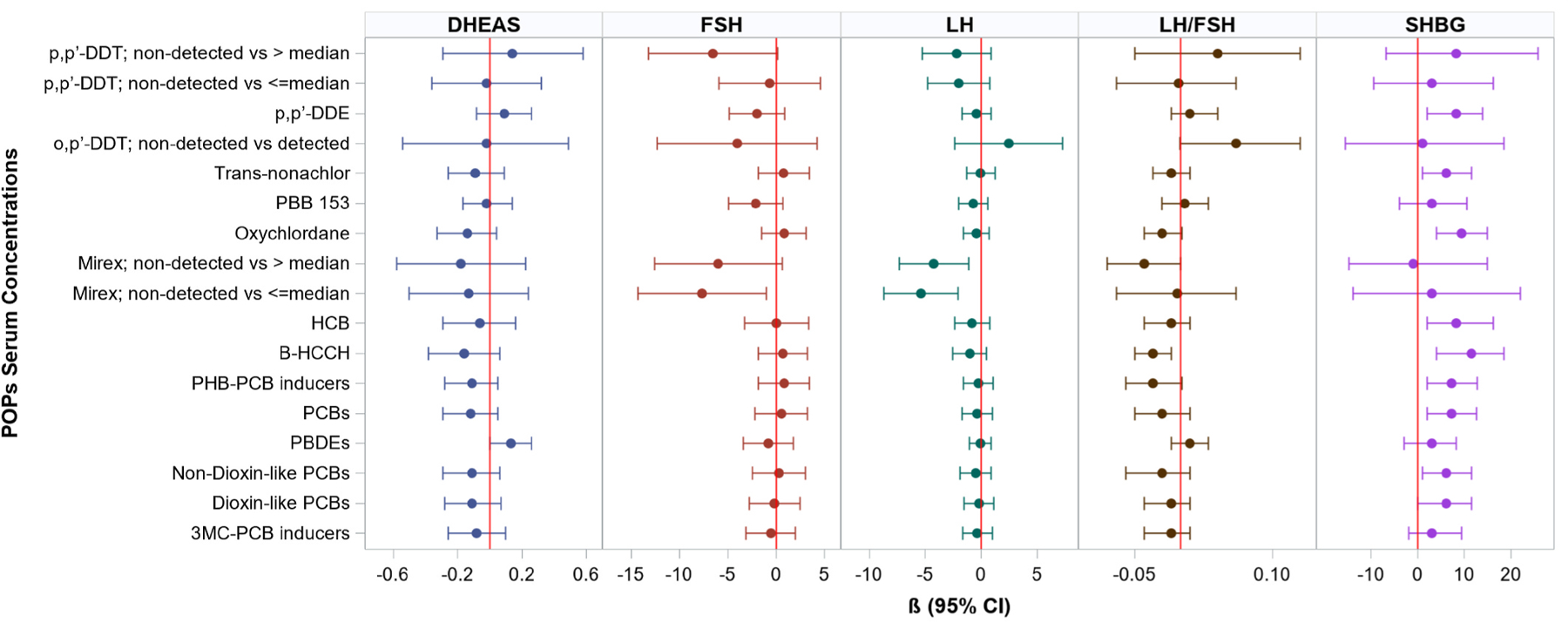
Adjusted β coefficients and 95% CI for associations of POPs serum concentrations (ng/g lipid) with sex-related hormones, Hispanic/Latino postmenopausal women (N = 716). Multivariable models adjusted for age, body mass index, waist-to-hip ratio, acculturation score Multiethnic Study of atherosclerosis, study sites, Hispanic/Latino background, educational attainment, estimated glomerular filtration rate, smoking status, alcohol consumption, physical activity levels, alternative healthy eating index 2010 and number of live births. Results correspond to data in Table S7. *Hormones*: luteinizing hormone (LH; mIU/mL), follicle stimulating hormone (FSH; mIU/mL), dehydroepiandrosterone sulfate (DHEAS; umol/L), and sex hormone binding globulin (SHBG; nmol/L). *POPs*: polybrominated diphenyl ethers (PBDEs), polychlorinated biphenyls (PCBs), (2-(4-chlorophenyl)-2-(2-chlorophenyl)-1,1,1-trichloroethane [o,p’-DDT], 1,1,1-trichloro-2,2-diphenylethane [p,p’-DDT], 1,1-dichloro-2,2-bis(p-chlorophenyl) ethylene [p,p’-DDE], polybrominated biphenyl (PBB), hexachlorobenzene [HCB], beta-hexachlorocyclohexane [β-HCCH], phenobarbital-type inducers-CYP IIB inducers (∑PHB-PCB inducers), and 3-methylcholanthrene inducers-CYP IA inducers/substrates (∑3 MC-PCB inducers).

Serum concentration of PCBs and OCPs were positively associated with SHBG (nmol/L) levels in postmenopausal women. A 1-quartile increase in ∑PCBs, ∑non-dioxin-like PCBs, ∑PHB-PCB inducers, ∑dioxin-like PCBs, β-HCCH, HCB, oxychlordane, *trans*-nonachlor, and p,p’-DDE was significantly associated with increased SHBG (nmol/L) levels (Fig. 1 and Table S7). Mirex concentrations above median (2.35 ng/g lipid) of detected values, and below or equal to median of detected values (reference = non-detected) were significantly associated with decreased LH levels (β = −4.23 mIU/mL, 95% CI: −7.33,−1.13) and (β = −5.36 mIU/mL, 95% CI: −8.67,−2.04; Fig. 1 and Table S7), respectively. Mirex concentration below or equal to median of detected values was also significantly associated with decreased levels of FSH (β = −7.66 mIU/mL, 95% CI: −14.3,−1.05; Fig. 1 and Table S7) whereas, a quartile increase in β-HCCH and oxychlordane concentrations were associated with decrease in the ratio of LH to FSH and p,p’-DDT concentrations below or equal to the median (8.59 ng/g lipid) of detected values (reference = non-detected) were associated with increased odds of having low E2 (OR = 2.64; 95% CI: 1.46,4.80; Table S7) in postmenopausal women. In addition, a quartile increase in ∑PBDEs was associated with borderline significant increase in DHEAS levels (β = 0.13 μmol/L, 95% CI: −0.001,0.26; Fig. 1 and Table S7). We did not observe evidence of significant non-linear associations of POPs serum concentrations with sex-related hormones in postmenopausal women (Table S8). Based on findings from previous studies, we conducted a sub-analyses evaluating associations of select PBDE congeners with DHEAS. In postmenopausal women, a quartile increase in PBDE-100 and PBDE-47 were associated with significant increase in DHEAS levels (β = 0.15 μmol/L, 95% CI: 0.01,0.28) and (β = 0.14 μmol/L, 95% CI: 0.02,0.27; Table S9), respectively.

#### 3.2.2. Association of POPs serum concentration with sex-related hormones in men

**Fig. 2.**
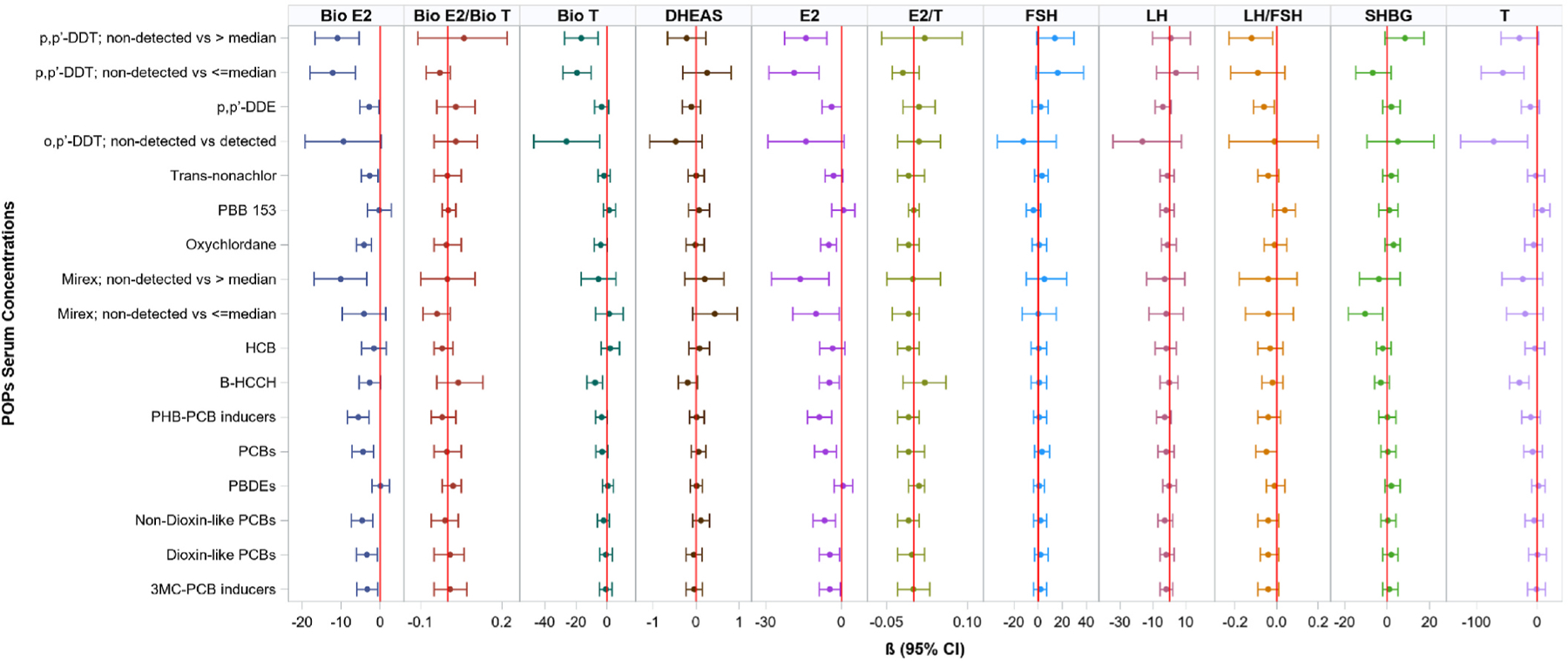
Adjusted β coefficients and 95% CI for associations of POPs serum concentrations (ng/g lipid) with sex-related hormones, Hispanic/Latino men (N = 1073). Multivariable models adjusted for age, body mass index, waist-to-hip ratio, acculturation score Multiethnic Study of atherosclerosis, study sites, Hispanic/Latino background, educational attainment, estimated glomerular filtration rate, smoking status, alcohol consumption, physical activity levels, and alternative healthy eating index 2010. Results correspond to data in Table S10. *Hormones*: luteinizing hormone (LH; mIU/mL), follicle stimulating hormone (FSH; mIU/mL), dehydroepiandrosterone sulfate (DHEAS; umol/L), sex hormone binding globulin (SHBG; nmol/L), estradiol (E2; pmol/L), testosterone (T; ng/dL), Bioavailable T (Bio T; ng/dL) and bioavailable E2 (bio E2; pmol/L). *POPs*: polybrominated diphenyl ethers (PBDEs), polychlorinated biphenyls (PCBs), (2-(4-chlorophenyl)-2-(2-chlorophenyl)-1,1,1-trichloroethane [o,p’-DDT], 1,1,1-trichloro-2,2-diphenylethane [p,p’-DDT], 1,1-dichloro-2,2-bis(p-chlorophenyl) ethylene [p,p’-DDE], polybrominated biphenyl (PBB), hexachlorobenzene [HCB], beta-hexachlorocyclohexane [β-HCCH], phenobarbital-type inducers-CYP IIB inducers (∑PHB-PCB inducers), and 3-methylcholanthrene inducers-CYP IA inducers/substrates (∑3 MC-PCB inducers).

Serum concentrations of PCBs and OCPs were inversely associated with E2 (pmol/L) and bioavailable E2 (pmol/L) levels in men. On average, a 1-quartile increase in ∑PCBs, ∑3 MC-PCB inducers, ∑dioxin-like PCBs, ∑non-dioxin-like PCBs, and ∑PHB-PCB inducers was significantly associated with decreased levels of E2 and bioavailable E2 (Fig. 2 and Table S10). Several OCPs, including β-HCCH, mirex, oxychlordane, *trans*-nonachlor, p,p’-DDE and p,p’-DDT were also associated with decreased levels of E2 and/or bioavailable E2. A quartile increase in p,p’-DDE (β = −0.06, 95% CI: −0.11,−0.01) and p,p’-DD T above than the median (4.04 ng/g lipid) of detected values (reference = non-detected; β = −0.12, 95% CI: −0.23,−0.02; Fig. 2 and Table S10) were associated with lower ratio of LH to FSH. A quartile increase in ∑PCBs was also associated with borderline significant lower ratio of LH to FSH. In addition, β-HCCH, p,p’-DDT, and o,p’-DDT were associated with decreased levels of T (ng/dL) and/or bioavailable T (ng/dL; Fig. 2 and Table S10). Our analysis did not show significant associations of POPs concentrations with LH, DHEAS, ratios of E2 to T, and bioavailable E2 to bioavailable T in men (Table S10). We also did not observe evidence of significant non-linear associations of POPs serum concentrations with sex-related hormones in men (Table S11).

Sensitivity analyses for association of POPs serum concentration with sex-related hormones outside the reference range in postmenopausal women and men.

Findings from sub-analyses evaluating associations of POPs serum concentration with sex-related hormones outside the reference range supported the main analysis using continuous sex hormones. In postmenopausal women, inverse associations were demonstrated for serum concentration of PCBs and OCPs with low SHBG, significant only for oxychlordane (Table S12). Serum concentrations of p,p’-DDE was also significantly associated with low DHEAS OR = 0.33, 95% CI: 0.13,0.81 (Table S12). In men, serum concentration of PCBs were significantly and inversely associated with high E2 but we found overall null associations for OCPs with high E2 (Table S13). Only oxychlordane OR = 0.55, 95% CI: 0.34,0.88 was significantly and inversely associated with low T (Table S13). We did not observe significant associations with high LH and FSH in men consistent with findings of continuous hormones.

Effect modification by adiposity on associations of POPs serum concentration with sex-related hormones in men and postmenopausal women.

Tables 2 and 3 present associations of POPs serum concentrations with sex-related hormones stratified by median BMI and waist-to-hip ratio in men and postmenopausal women. Overall, in men with BMI below the median value of 28.1 kg/m^2^, serum concentrations of select POPs were associated with lower ratio of LH to FSH, LH, SHBG, T, and bioavailable T. Conversely, in men with BMI above 28.1 kg/m^2^, the associations tended to be positive (Table 2). Likewise, POPs were associated with lower E2 and bioavailable E2 levels in men with waist-to-hip ratio below the median value of 0.96 and associated with higher levels in men with waist-to-hip ratio above the median (Table 3). In postmenopausal women, effect modification by BMI and waist-to hip ratio on associations of POPs serum concentration with sex-related hormones showed inconsistent findings (Tables 2 and 3).

## 4. Discussion

To our knowledge, this is one of the largest studies to date evaluating the relationship between POPs and sex-related hormones in both middle aged and older men as well as postmenopausal women. It is also the first study to explore these relationships in a predominantly immigrant, heterogeneous Hispanic/Latino adult population with higher body burden of pesticides and disproportionate exposure to environmental contaminants in the U.S. Overall, we found consistent associations of serum concentrations of PCBs and OCPs with decreased levels of E2 and bioavailable E2 in men and increased levels of SHBG in postmenopausal women. β-HCCH, p,p’-DDT, and o,p’-DDT were associated with decreased levels of T and bioavailable T in men, and ∑PBDEs, PBDE-100 and PBDE-47 congeners were associated with increased DHEAS levels in postmenopausal women. We also observed evidence of effect modification by adiposity primarily in men. Serum concentrations of PCBs and OCPs were inversely associated with select sex-related hormones in men with BMI below 28.1 kg/m^2^ and positively associated in men with BMI greater 28.1 kg/m^2^. Specifically, stronger inverse associations of PCBs, PBDEs, and several OCPs were observed with LH, SHBG, E2, bioavailable E2, T, and the ratios of LH to FSH and E2 to T in men with BMI below 28.1 kg/m^2^. Our findings are particularly compelling given the aging Hispanic/Latino population in the U.S. and the existing disparities in cardiometabolic disease risk which may be further exacerbated by the present results.

### 4.1. Comparison of POPs serum concentration with those in other studies

Prior analysis of data from the U.S. population using the National Health and Nutrition Examination Survey (NHANES) did not demonstrate racial or ethnic differences in PBDE concentrations (Sjödin et al., 2014). Previous analysis compared POPs concentration in the current HCHS/SOL ancillary study to those in Hispanics aged 40 years and older in the 2009–2010 NHANES cycle (Day, 2022). Overall, the majority of POPs exhibited lower serum concentrations among HCHS/SOL participants compared to NHANES Hispanic participants except HCB which was present at higher levels particularly among HCHS/SOL participants of Mexican descent (Day, 2022). We also present comparisons to other studies, noting potential limitations related to occupational exposure, age range of participants, lipid standardization and the units of POPs measurement reported. We observed lower concentrations of ΣPBDEs for HCHS/SOL men when compared to men in the Great Lakes Fish Study (Turyk et al., 2008), similar concentrations when compared to men in the Flame Retardant Exposure Study (Makey et al., 2016), and higher concentrations of ΣPBDEs for postmenopausal women in this study than those reported for Chinese women in the Laizhou Wan (Bay) Birth Cohort study) (Gao et al., 2016).

Additionally, we observed higher concentrations of p,p’-DDE but lower concentrations of HCB and ∑PCBs for men in the present ancillary study compared to male partners in subfertile couples treated at Massachusetts General Hospital (Ferguson et al., 2012). Lower concentrations of ∑DL-PCBs and HCB but higher concentration of ∑NDL-PCBs, p, p’-DDE and β-HCCH were observed when comparing postmenopausal women in the present study to Chinese women (Pan et al., 2019). Differences in findings among published studies may relate to several factors, including variations in the assessment and exclusion of individuals using medications impacting hormones, effect of chemical mixtures and unmeasured chemical exposures that may impact sex hormone homeostasis, varying concentrations and types of POPs measured across the different studies, and differences in biomarker detection methods, study design, and characteristics of study participants (Freire et al., 2014; Tomczak et al., 1981; Cohn et al., 2007). Our study population also comprised recent immigrants with prolonged exposure to OCPs outside the USA along with varying diet-related and socio-cultural factors and exposure to chemicals within the borders of the USA. These may have also influenced POPs concentration in our study.

### 4.2. Associations of PBDEs with sex-related hormones: comparisons with other studies and biological mechanisms

In women, exposure to PBDEs was associated with disruption of reproductive outcomes, including preterm birth and age at menarche (Peltier et al., 2015; Chen et al., 2011); however, studies in post-menopausal women are limited. In men, PBDE was inversely associated with sperm mobility (Abdelouahab et al., 2011). We found increased levels of DHEAS with increasing quartiles of ∑PBDEs, PBDE-100 and PBDE-47 serum concentrations in postmenopausal women. Previous studies evaluating these relationships with DHEAS in women are limited. One study of pregnant Chinese women found inverse associations of PBDE with FSH but not LH (Gao et al., 2016). Mixed and congener-specific associations have also been reported in the association of PBDEs with LH, FSH, and T in boys and men (Johnson et al., 2013; Turyk et al., 2008; Makey et al., 2016; Eskenazi et al., 2017). While evidence demonstrates that brominated flame retardants [PBDEs and PBB 153] may disrupt thyroid homeostasis (Yeshoua et al., 2024), effects on reproductive toxicity and dysregulation of sex-related hormones remains inconclusive. Current hypotheses suggests that PBDEs may exert both agonistic and antagonistic effects on hormones without activating the AhR pathway (Ezechiáš et al., 2012; Legler and Brouwer, 2003).

In the present study, ΣPBDEs, PBDE-100 and PBDE-47 were associated with increased DHEAS levels in postmenopausal women which contrasts with previous experimental studies suggesting that PBDEs may inhibit DHEA synthesis through the CYP17A1 (17α-hydroxylase) - a key enzyme in the steroidogenic pathway (Cantón et al., 2006; Song et al., 2008). However, effects of PBDEs on sulfotransferase enzymes, which are critical for sulfation and conversion of DHEA to DHEAS in the adrenal cortex, remain poorly understood highlighting the need for further research. While we did not evaluate T and progesterone levels in postmenopausal women in the present study due to limited sample volume, DHEAS is a precursor for androgen and estrogen synthesis and findings of increased levels may be important in elucidating underlying mechanisms involved in the relationship of exposure to PBDEs with sex-steroid dysregulation. Associations of PBDEs with E2 above or below the LOD were null in postmenopausal women and differed from experimental studies conducted in animal models demonstrating increased secretion of T, estrogen, and progesterone from theca and granulosa cells of porcine ovarian follicles (Gregoraszczuk et al., 2008; Karpeta and Gregoraszczuk, 2010). However, comparisons with animal models have inherent limitations and we could not evaluate E2 as a continuous outcome in women given that 71% of women in our study had E2 levels below the reference range which could have also impacted observed associations.

### 4.3. Associations of OCPs and PCBs with sex-related hormones: comparisons with other studies and biological mechanisms

We also found increased levels of SHBG with increasing serum concentration of various OCPs and PCB groups in postmenopausal women. Few studies have evaluated the relationship of POPs with SHBG in women, with only one study using cord blood samples reporting increased SHBG levels with PCBs in female newborns (Warembourg et al., 2016). Furthermore, previous investigations in men reported mixed associations of POPs with E2 and bioavailable E2. Consistent with our findings, decreased levels of E2 and T with exposure to PCBs (Goncharov et al., 2009; Turyk et al., 2006) and select OCPs (Freire et al., 2014; Blanco-Muñoz et al., 2012; Martin et al., 2002; Ayotte et al., 2001) were reported in some studies. In contrast, others have found null associations of POPs exposure with E2 and T (Persky et al., 2001; Langer et al., 2010; Goncharov et al., 2009), while some positive associations have also been reported (Bornman et al., 2018; Dalvie et al., 2004).

There are several possible mechanisms that could explain the associations we observed of OCPs and PCBs with sex-related hormones. The effect of POPs on sex hormone dysregulation may be mediated through the disruption of pathways involved in the synthesis, metabolism, and regulation of androgenic and estrogenic hormones (Sharpe and Irvine, 2004; Vested et al., 2014; Wahlang, 2018). More specifically, estrogenic PCBs suppress hepcidin, disrupt iron homeostasis, and alter lipid metabolism and hormone synthesis, while dioxin-like-PCBs demonstrate anti-estrogenic properties inhibiting estrogen receptor (ER) signaling pathways, and binding of E2 to ER (Warner et al., 2012; Qian et al., 2015; Cooke et al., 2008; Wolff et al., 1997; Eske et al., 2014; Guillotin and Delcourt, 2022). Coplanar anti-estrogenic and highly chlorinated PCBs also activate the aryl hydrocarbon receptor (AhR), constitutive androstane and pregnane xenobiotic receptors, and the cytochrome P450, family 1, subfamily A, polypeptide 1 (CYP1A1) genes, thereby stimulating the production of cyclooxygenase (COX-2) and reactive oxygen species resulting in oxidative stress, genotoxicity, and dysregulation of endocrine and metabolic function (Warner et al., 2012; Parker et al., 2018; Safe et al., 1998). Experimental studies also provide evidence that other POPs exhibit both androgenic and estrogenic activity (Kelce et al., 1995; Burgos-Aceves et al., 2021; Iwasaki et al., 2023; Scippo et al., 2004; Ralph et al., 2003; Faroon et al., 1995).

In the present study, non-dioxin-like (estrogenic) and dioxin-like (non-estrogenic) PCBs demonstrated similar associations with SHBG in postmenopausal women, and with E2, bioavailable E2, T, and bioavailable T in men. The precise mechanisms through which POPs influence SHBG levels in postmenopausal women, and E2 and T levels in men remain unclear and requires further investigation. POPs may impact mechanisms involved in the synthesis and release of SHBG, by potentially binding directly to SHBG and/or its receptors (Hong et al., 2016; Le et al., 2012), interfering with hormonal signaling pathways that regulate SHBG production, influencing SHBG-related gene expression or altering liver function and SHBG synthesis (Danzo, 1997; Pugeat et al., 2010). SHBG binds to androgens and estrogens, thereby influencing their bioavailability with evidence from the present HCHS/SOL cohort demonstrating inverse associations of SHBG with diabetes and glycemic traits in postmenopausal women, although specific pathways remain unclear (Persky et al., 2023).

Specific POPs including PCBs and OCPs may also induce crosstalk between AhR and ER, upregulating genes related to aromatase activity such as cytochrome 19 (CY19) genes, and inhibiting estradiol sulfation, potentially impacting SHBG levels and estrogen activity (Warner et al., 2012; Kojima et al., 2004). 17α-hydroxylase, a key enzyme in androgen biosynthesis may also be inhibited by exposure to PCBs and OCPs (Warner et al., 2012; Kojima et al., 2004; Vasaitis et al., 2011). Taking together, these mechanisms could help explain the present findings of decreased levels of T, bioavailable T, E2 and bioavailable E2 in men. These findings are significant given that T and estrogen are important clinical biomarkers with implications for quality of life and downstream health outcomes in both men and women including diabetes, hypertension, cardiovascular health, osteoporosis and bone fracture (Persky et al., 2023; Shigehara et al., 2021).

In women, increasing concentration of β-HCCH and oxychlordane, and in men, p,p’-DDE and p,p’-DDT concentrations above the median of 4.04 ng/g lipid were associated with a decreased ratio of LH to FSH. Prior studies indicate that increased ratio of LH to FSH is associated with polycystic ovary syndrome in women characterized by decreased ovarian reserve and moderate elevations in T (Yang and Chen, 2024). In men, normal or declining LH and/or FSH levels have been linked to declining testosterone levels (Fernandez et al., 2019) while in animal models, E2 increased LH and decreased FSH levels (Wang et al., 2021). While it is not fully understood, the present findings of inverse associations for ratio of LH to FSH with increasing levels of select OCPs in men and postmenopausal women as well as declining T levels in men support mechanisms suggesting antiandrogenic activity of OCPs particularly chlordane, DDE and DDT (Kojima et al., 2004). Findings of decreased ratio of LH to FSH also support direct inhibition of estrogen signaling in the pituitary (Wang et al., 2021) consistent with results of inverse associations of OCPs with E2 and bioavailable E2 in men. POPs may also disrupt gonadotropin-releasing hormone pulse generator and other signaling factors including activins, inhibins, and follistatins which differentially regulate FSH but not LH secretion (Wang et al., 2021; Welt et al., 2002). However, these mechanisms are currently underexplored in previous investigations. Our findings are compelling since the Hispanic/Latino population are comprised of recent immigrants with exposure to OCPs outside the USA, ongoing present-day exposure to chemicals within the borders of the USA, and elevated risk of cardiometabolic diseases.

### 4.4. Effect modification with measures of adiposity and biological mechanisms

We also observed evidence of possible effect modification with PCBs, PBDEs, and several OCPs inversely associated with LH, SHBG, E2, bioavailable E2, T, and the ratios of LH to FSH and E2 to T in men with BMI below 28.1 kg/m^2^ and positively associated in men with BMI above 28.1 kg/m^2^. However, the role of adiposity on associations of POPs with sex-related hormones has not been thoroughly investigated in many studies. To our knowledge, only one study observed inverse associations of heptachlor epoxide with SHBG when BMI was below 25 kg/m^2^, but no associations when BMI was above 25 kg/m^2^ in men (Madrigal et al., 2021).

The biological pathways through which adiposity modifies associations between POPs and sex-related hormones are not fully elucidated although some hypotheses have been proposed relating to the interplay between adipose tissues and the liver in regulating endocrine function, energy homeostasis, and POPs metabolism (Law et al., 2014; Koceva et al., 2024; Pott et al., 2021; Arsenescu et al., 2008; Chevrier et al., 2000; Dirinck et al., 2011; Wolff et al., 2007; Aaseth et al., 2022; Rhyu and Yu, 2021; Kumar et al., 2014). Postmenopausal decreases in estrogen are linked to increased body fat due to reduced energy expenditure; declining testosterone in men is associated with increased fat mass; and obesity-related hypogonadism affects both sexes (Law et al., 2014; Pott et al., 2021; Eng et al., 2024). Moreover, steroid hormone signaling and conversion enzymes are important for adipose tissue regulation (Law et al., 2014; Pott et al., 2021). Epidemiological studies on the relationship of POPs with adiposity measures are inconclusive and cross-sectional investigations can be strongly biased by the long-term accumulation of POPS in lipid tissues (Chevrier et al., 2000; Dirinck et al., 2011; Wolff et al., 2007; Aaseth et al., 2022). Weight loss can also result in POPs redistribution and increased serum concentrations potentially heightening toxic effects on target organs (Arsenescu et al., 2008; Chevrier et al., 2000; Dirinck et al., 2011; Wolff et al., 2007; Aaseth et al., 2022).

Both experimental and epidemiological studies provide evidence that POPs disrupt adipose tissue metabolism and liver homeostasis, increase biomarkers of liver dysfunction and proinflammatory activity in adipose tissues, and upregulate genes involved in adipogenesis through increased expression of peroxisome proliferator-activated receptor γ, CCAAT/enhancer-binding protein alpha and leptin (Law et al., 2014; Pott et al., 2021; Aaseth et al., 2022; Rhyu and Yu, 2021; Kumar et al., 2014). Lipid metabolism differs by BMI and may impact sex steroid metabolism (Palmisano et al., 2018), although the role of POPs in this pathway is not fully elucidated. Fat depots around the liver also differs by obesity status and may influence both POPs and sex steroid metabolism (Aaseth et al., 2022; Rhyu and Yu, 2021; Kumar et al., 2014), however, this has not been well studied. Taken together, these alterations may differentially impact sex hormone dysregulation across measures of adiposity warranting further investigation in future research.

### 4.5. Limitations and strengths

The present study is subject to some limitations. First, results may not be generalizable to other racial and ethnic groups given the exposure, dietary and sociocultural profile within the Hispanic/Latino population. Second, POPs serum concentrations and sex-related hormones were measured at a single timepoint hence findings may not capture an individual’s hormone or POPs concentration across time. However, a hallmark of POPs exposures is the long elimination half-lives (up to 10 years or longer), so a single measurement may adequately represent past and recent exposures. Third, we were unable to evaluate associations of POPs with E2 and T in women due to inadequate sample volume for the measurement of T and measured E2 concentrations below the LOD in 71% of samples. Fourth, HCHS/SOL assessed information on covariates such as smoking, alcohol use and education by self-report which may be subject to recall bias. Fifth, we did not account for multiple comparisons given that our hypotheses were prespecified. However, there is the possibility of false positive findings due to the large number of comparisons emphasizing the need to replicate these results in future studies of other populations (Goldberg and Silbergeld, 2011). Sixth, the present analysis did not evaluate associations of POPs serum concentrations with sex-related hormones using mixtures methodology, but this should be investigated in future studies. Seventh, despite controlling for a wide range of confounders, residual confounding may be present due to exposure to unmeasured pollutants and factors related to POPs and hormones including percent body fat, body fat distribution, systemic inflammation, hepatic function and hepatic adipose content.

Eight, we did not include information on current or historical employment since comprehensive data on industries and sectors, and work history were not available for this population; however, this should be addressed in future epidemiological studies. Ninth, although there is demonstrated heterogeneity within Hispanic/Latino subgroups in cardiometabolic disease burden (Elfassy et al., 2020; Daviglus et al., 2012), sex-related hormone levels (Abasilim et al., 2023), pesticide use, restrictions and bans (UNEP United Nations Environment Programme and Chemicals, 2002a; UNEP United Nations Environment Programme and Chemicals, 2002b), and POPs profiles (Day, 2022), we are limited by sample size, and cannot combine Hispanic/Latino subgroups for stratified analysis given the HCHS/SOL survey design, sampling strategy and investigator analytical guideline. Multivariable models, however, control for confounding on associations of POPs with sex hormones by Hispanic/Latino background. Tenth, we do not specifically control for fish consumption in the present study due to imprecise dietary variables and lack of detailed data on fish types consumed. Moreover, fish consumption only predicted ∑PCBs concentrations among men in previous analyses (Day, 2022). Models, however, control for alternative heathy eating index given that other dietary components can influence sex-related hormones. Eleventh, our data on the type of menopause did not distinguish between hysterectomy alone versus hysterectomy with oophorectomy which limits our ability to categorize postmenopausal women with surgical menopause. Hence we could not evaluate whether type of menopause modified associations between POPs and sex-related hormone levels in postmenopausal women.

Our study has several important strengths. First, the present HCHS/SOL ancillary study is unique in assessing associations of POPs serum concentrations with sex-related hormones in a heterogenous Hispanic/Latino population. Secondly, we carefully evaluated menopause status in women using clinical data and concentrations of endogenous sex-related hormone. We also extensively reviewed medication use under an endocrinologist’s guidance, excluding individuals using hormone-related medications. Third, we examined biologically plausible pathways through which adiposity may impact associations of POPs serum concentration with sex-related hormones. Fourth, we maintained a robust sample size by applying multiple imputation methodology which prevented loss of information and sufficient statistical power to identify observed effects. Fifth, our analysis evaluating associations of PCB serum concentration with sex-related hormones used established groups that account for biological, pharmacokinetic, and structural mechanisms (Phillips et al., 1989; Warner et al., 2012).

## 5. Conclusions

Our findings suggest that exposure to PCBs and OCPs were associated with decreased levels of E2, bioavailable E2, T, and bioavailable T in men as well as increased levels of SHBG in postmenopausal women. We also observed evidence suggesting that associations of POPs with sex-related hormones were modified by adiposity, as measured by body mass index and waist-to-hip ratio. Effect modification by adiposity was more pronounced in men than in postmenopausal women. Our findings are significant given the role of hormonal dysregulation in cardiometabolic disease development and the greater exposure to endocrine disrupting chemicals and elevated burden of cardiometabolic diseases in the U.S. Hispanic/Latino population. Further research into how adiposity influences the relationship between POPs and sex-related hormones could inform interventions such as weight management, aimed at preventing endocrine-related diseases particularly diabetes and hypertension. Given that hormonal disruption following POPs exposure involves multiple pathways that may vary by sex, additional studies are warranted to further elucidate these relationships which may be more pronounced in understudied minority populations.

## Supplementary Material

1

## Figures and Tables

**Table 1:** Distribution of POPs concentrations (ng/g lipid) and sociodemographic, clinical and lifestyle characteristics at baseline examination (V1) in Hispanic/Latino men and postmenopausal women.

	Postmenopausal women (n = 716)	Men (n = 1073)
**∑PBDEs; geometric mean (95% CI)**	24.2 [20.8,28.1]	29.3 [27.5,31.2]
**∑PCBs; geometric mean (95% CI)**	129.8 [118.4142.2]	122.1 [114.9129.7]
**∑3 MC-PCB inducers; geometric mean (95% CI)**	19.9 [17.9,22.2]	13.6 [12.6,14.8]
**∑Dioxin-like PCBs; geometric mean (95% CI)**	16.1 [14.5,17.9]	11.8 [11.0,12.8]
**∑Non-Dioxin-like PCBs; geometric mean (95% CI)**	112.9 [103.1123.7]	109.6 [103.3116.3]
**∑PHB-PCB inducers; geometric mean (95% CI)**	74.1 [67.8,81.0]	75.6 [71.3,80.2]
**PBB 153; geometric mean (95% CI)**	1.02 [0.85,1.24]	1.31 [1.15,1.50]
**β-HCCH; geometric mean (95% CI)**	13.9 [11.6,16.6]	5.51 [4.78,6.36]
**HCB; geometric mean (95% CI)**	14.7 [13.5,16.0]	11.4 [10.4,12.4]
**Oxychlordane; geometric mean (95% CI)**	10.9 [9.66,12.2]	6.78 [6.40,7.18]
**Trans-nonachlor; geometric mean (95% CI)**	16.2 [14.2,18.4]	11.9 [11.3,12.5]
**p,p’-DDE; geometric mean (95% CI)**	788.3 [695.8893.2]	386.5 [342.2436.6]
**o,p’-DDT; n (%)**		
Non-detected	666 (93)	1010 (93)
Detected	50 (7)	63 (7)
**Mirex; n (%)**		
Non-detected	381 (52)	413 (38)
≤ Median of detected values	168 (24)	334 (29)
> Median of detected values	167 (24)	326 (33)
**p,p’-DDT; n (%)**		
Non-detected	342 (45)	615 (55)
≤ Median of detected values	188 (25)	219 (22)
> Median of detected values	186 (29)	235 (23)
**Age in years; mean (95% CI)**	59.3 [57.9,60.7]	55.5 [54.6,56.3]
Age 45–54	281 (30)	640 (50)
Age 55–64	352 (41)	331 (32)
Age 65+	83 (29)	102 (18)
**Hispanic/Latino background; n (%)**		
Dominican	84 (9.7)	89 (8.5)
Central American	75 (6.5)	94 (6.8)
Cuban	101 (28)	218 (29)
Mexican (reference)	266 (31)	386 (31)
Puerto Rican	129 (17)	173 (15)
South American	47 (4.3)	86 (4.5)
More than one/Other heritage	14 (3.2)	27 (4.9)
**Educational attainment; n (%)**		
Less than high school	326 (40)	384 (36)
High school diploma/GED	137 (17)	261 (22)
Greater than high school diploma	253 (43)	428 (43)
**Body mass index [kg/m^2^]; mean (95% CI)**	29.9 [29.0,30.7]	28.1 [27.7,28.6]
Under/normal weight (BMI <25)	125 (19)	223 (23)
Overweight (25 ≤ BMI <30)	271 (40)	508 (47)
Obese (BMI ≥30)	320 (41)	342 (30)
**Physical activity level; n (%)**		
High	36 (4.1)	186 (15)
Moderate	301 (43)	495 (45)
Low	379 (53)	392 (40)
**Cigarette use; n (%)**		
Never (reference)	455 (67)	439 (39)
Former	145 (17)	359 (35)
Current	116 (16)	275 (27)
**Alcohol use; n (%)**		
None	445 (65)	426 (44)
Low	258 (33)	558 (49)
High	13 (1.4)	89 (7.2)
**Field center; n (%)**		
Bronx	184 (29)	222 (26)
Chicago (reference)	157 (11)	283 (14)
Miami	176 (38)	321 (38)
San Diego	199 (22)	247 (23)
**Prediabetes status; n (%)**		
Normoglycemic	317 (42)	549 (35)
Prediabetic	399 (58)	524 (65)
**Acculturation score – MESA; mean (95% CI)**	1.78 [1.59,1.97]	1.80 [1.66,1.93]
**AHEI-2010; mean (95% CI)**	50 [49.1,50.9]	51.5 [50.8,52.2]
**Waist to Hip Ratio; mean (95% CI)**	0.90 [0.89,0.91]	0.96 [0.96,0.97]
**eGFR; mean (95% CI)**	90.5 [88.4,92.6]	94.3 [92.7,95.9]
**Number of prior live births (females only); n (%)**		
0–1	121 (18)	–
2	199 (32)	–
3–4	294 (35)	–
5+	102 (15)	–

Distribution of POPs concentrations and characteristics of participants were stratified by subgroups of sex; hence differences were not evaluated using statistical tests.

∑PBDEs — PBDE 100, 153, 154, 17, 183, 209, 28, 47, 85, 99.

∑PCBs — PCB 105, 114, 118, 138–158, 156, 153, 156, 157, 167, 170, 178, 180, 183, 187, 189, 194, 196–203, 199, 206, 209, 28, 66, 74, 99.

∑3-methylcholanthrene (3 MC) inducers: CYP IA inducers/substrates – PCB 66, 74, 105, 118, 156, 167.

∑Dioxin-like PCBs — PCB 105, 114, 118, 156, 157, 167, 189.

∑Non-Dioxin-like PCBs — PCB 28, 66, 74, 99, 138158, 146, 153, 170, 178, 180, 183, 187, 194, 196–203, 199, 206, 209.

∑Phenobarbital-type (PB) inducers: CYP IIB inducers – PCB 99, 153, 180, 183, 187, 194, 196–203, 199, 206.

**Table 2:** Associations of POPs serum concentrations (ng/g lipid) with sex-related hormones ^a^ in Hispanic/Latino men and postmenopausal women, stratified on median body mass index.

POPs → Hormones	Below median BMI β (95% CI)	Above or equal to median BMI β (95% CI)
Men (N = 1073)		
∑3 MC-PCB inducers → LH^b^	−4.88 [−10.4,−0.1]**	3.05 [−1.98,9.42]
∑DL-PCBs → LH^b^	−4.88 [−9.52,1.01]	3.05 [−2.96,8.33]
∑PBDEs → SHBG^b^	−0.10 [−3.92,4.08]	3.05 [−2.96,8.33]
Mirex→ SHBG;^b,c^ non-detected vs ≤ median	−2.96 [−13.9,9.42]	−14.8 [−23.7,−4.88]**
non-detected vs > median	5.13 [−5.82,18.5]	−10.4 [−22.1,3.05]
HCB → SHBG^b^	−5.82 [−9.52,−1.00]**	3.05 [−2.96,8.33]
∑PBDEs → DHEAS	0.12 [−0.08,0.31]	3.05 [−1.98,9.42]
∑PBDEs → T	−8.14 [−23.7,7.45]	5.86 [−9.09,20.8]
∑3 MC-PCB inducers → T	−19.8 [−40.6,0.99]	13.3 [−3.00,29.6]
∑DL-PCBs → T	−17.2 [−38.5,4.07]	12.9 [−2.89,28.7]
β-HCCH → T	−44.9 [−66.4,−23.5]**	−19.3 [−39.7,1.00]
HCB → T	−20.1 [−42.9,2.70]	5.21 [−15.8,26.2]
p,p’-DDE → T	−29.3 [−51.5,−7.14]**	−2.42 [−19.5,14.6]
∑PCBs → Bio T	−4.69 [−10.2,0.80]	−0.82 [−6.26,4.62]
∑3 MC-PCB inducers → Bio T	−4.73 [−10.1,0.61]	2.06 [−3.08,7.20]
∑DL-PCBs → Bio T	−4.95 [−10.4,0.45]	2.74 [−2.33,7.80]
∑NDL-PCBs → Bio T	−4.48 [−9.99,1.04]	1.02 [−4.96,7.00]
∑PCBs → LH/FSH ratio	−0.09 [−0.16,−0.02]**	0.02 [−0.06,0.09]
∑3 MC-PCB inducers → LH/FSH Ratio	−0.10 [−0.17,−0.03]**	0.05 [−0.02,0.12]
∑DL-PCBs → LH/FSH ratio	−0.08 [−0.15,−0.01]**	0.03 [−0.03,0.10]
∑NDL-PCBs → LH/FSH ratio	−0.08 [−0.15,−0.01]**	0.03 [−0.04,0.10]
∑PHB-PCB inducers → LH/FSH ratio	−0.08 [−0.15,−0.01]**	0.04 [−0.03,0.11]
Oxychlordane → LH/FSH ratio	−0.04 [−0.11,0.02]	0.02 [−0.05,0.09]
Trans-nonachlor → LH/FSH ratio	−0.08 [−0.14,−0.02]**	0.001 [−0.07,0.08]
p,p’-DDT→ LH/FSH ratio;^c^ non-detected vs ≤ median	−0.10 [−0.29,0.08]	−0.02 [−0.16,0.12]
non-detected vs > median	−0.19 [−0.33,−0.04]**	−0.06 [−0.23,0.11]
∑NDL-PCBs → E2/T ratio	0.01 [−0.02,0.04]	−0.03 [−0.05,−0.01]**
∑PHB-PCB inducers → E2/T ratio	0.004 [−0.03,0.04]	−0.04 [−0.06,−0.02]**
Postmenopausal women (N = 716)		
β-HCCH → FSH	1.98 [−1.71,5.66]	−2.38 [−6.14,1.39]
Oxychlordane → FSH	1.43 [−1.78,4.64]	1.93 [−1.45,5.30]
p,p’-DDE → SHBG^b^	5.13 [−1,10.52]	9.42 [1.01,18.53]**
Mirex → DHEAS;^c^ non-detected vs ≤ median	−0.42 [−0.87,0.04]	0.12 [−0.43,0.67]
non-detected vs > median	−0.03 [−0.54,0.47]	−0.48 [−1.08,0.12]

When product terms suggested effect modification (p < 0.05), we stratified models by median BMI of 28.1 kg/m^2^ in men and 29.2 kg/m^2^ in postmenopausal women.

** p < 0.05.

a Multivariable models adjusted for age, body mass index, waist-to-hip ratio, acculturation score Multiethnic Study of atherosclerosis, study sites, Hispanic/Latino background, educational attainment, estimated glomerular filtration rate, smoking status, alcohol consumption, physical activity levels, alternative healthy eating index 2010 and number of live births (women only).

b Ln transformed SHBG was modeled in men and women, while Ln transformed LH and FSH were modeled in men. Estimates for associations of POPs serum concentration with Ln transformed hormones were back transformed [100 (*e^βPOPS^* −1)%] and can be interpreted as a quartile increase in POPs concentration is associated with relative change in median or geometric mean of hormone corresponding to β%.

c Mirex and p,p’-DDT were categorized as non-detected, below or equal to median of detected values, and above median of detected values. Other POPs were evaluated in quartiles and interpreted as per quartile increase in concentration.

**Table 3:** Associations of POPs serum concentrations (ng/g lipid) with sex-related hormones ^a^ in Hispanic/Latino men and postmenopausal women, stratified on median waist-to-hip ratio.

POPs → Hormones	Below median waist-hip-ratio β (95% CI)	Above or equal to median waist-hip-ratio β (95% CI)
**Men (N** = **1073)**		
**∑PBDEs** → **L**H^b^	−1.98 [−6.76,2.02]	3.05 [−1.98,8.33]
**PBB 153** → **L**H^b^	2.02 [−3.92,7.25]	−5.82 [−11.3,1.01]
**∑PBDEs** → **E2**	−2.34 [−6.15,1.47]	3.69 [−1.78,9.16]
**β-HCCH** → **E2**	−8.07 [−12.5,−3.69]**	0.15 [−6.77,7.07]
**β-HCCH** → **Bio E2**	−4.79 [−7.52,−2.07]**	0.01 [−4.97,4.99]
**∑PBDEs** → **T**	−6.45 [−20.9,8.00]	9.87 [−4.93,24.7]
**Mirex**→ **T**;^c^ non-detected vs ≤ median	−6.44 [−46.7,33.8]	−31.8 [−71.2,7.62]
non-detected vs > median	−8.31 [−50.2,33.6]	−47.0 [−95.4,1.44]
**HCB** → **E2/T ratio**	−0.01 [−0.02,0.01]	−0.02 [−0.04,−0.001]**
**Postmenopausal women (N** = **716)**		
**∑PBDEs** → **LH**	−0.83 [−2.31,0.64]	1.10 [−0.07,2.26]
**∑PBDEs** → **FSH**	−2.02 [−5.77,1.73]	0.23 [−2.30,2.75]
**PBB 153** → **FSH**	−3.51 [−7.40,0.38]	−0.58 [−3.99,2.83]
**Oxychlordane** → **SHB**G^b^	16.2 [8.33,23.4]**	2.02 [−3.92,8.33]
**Mirex** → **DHEAS**;^c^ non-detected vs ≤ median	0.30 [−0.21, 0.80]	−0.60 [−1.14, −0.06]**
non-detected vs > median	0.20 [−0.27, 0.66]	−0.72 [−1.31, −0.12]**
**PBB 153** → **LH/FSH ratio**	0.02 [−0.01,0.04]	−0.02 [−0.05,0.01]

When product terms suggested effect modification (p < 0.05), we stratified models by median waist-hip-ratio of 0.96 in men and 0.91 in postmenopausal women.

** p < 0.05.

a Multivariable models adjusted for age, body mass index, waist-to-hip ratio, acculturation score Multiethnic Study of atherosclerosis, study sites, Hispanic/Latino background, educational attainment, estimated glomerular filtration rate, smoking status, alcohol consumption, physical activity levels, and alternative healthy eating index 2010.

b Ln transformed SHBG was modeled in men and women, while Ln transformed LH and FSH were modeled in men. Estimates for associations of POPs serum concentration with Ln transformed hormones were back transformed [100 e^βPOPS^ −1)%] and can be interpreted as a quartile increase in POPs concentration is associated with relative change in median or geometric mean of hormone corresponding to β%.

c Mirex was categorized as non-detected, below or equal to median of detected values, and above median of detected values. Other POPs were evaluated in quartiles and interpreted as per quartile increase in concentration.

## Data Availability

Data will be made available on request.

## Appendix A. Supplementary data

Supplementary data to this article can be found online at https://doi.org/10.1016/j.envres.2024.120742.
