# Supplementary material for: Persistent organic pollutants and endogenous sex-related hormones in Hispanic/Latino adults: The Hispanic Community health study/study of Latinos (HCHS/SOL)
Source: Environ Res. Author manuscript; Available in PMC 2025 Nov 6. (PMC12591841; doi:10.1016/j.envres.2024.120742)
Supplement: 1 [file NIHMS2119849-supplement-1.docx]

**Appendix S1**

**Methods**

**Sensitivity analyses including only PCB and PBDE congeners with detection frequency thresholds above 60% and 75%**

We excluded congeners from summed PCB and PBDE groups if the detection frequencies were below 60% and 75%, respectively. For the analysis evaluating congeners demonstrating detection frequency above 60%, we excluded PCB congener 189 and PBDE congeners 154, 17, 183 and 85. The analysis evaluating congeners with detection frequency below 75% excluded PCB congeners 189, 66, 114 and PBDE congeners 209, 154, 17, 183 and 85. We did not find substantial differences in estimates of associations between PCB and PBDE groups with sex-related hormones in the sensitivity analysis excluding congeners as described above when compared to the primary analysis.

**Figure S1. Directed acyclic graph** **showing causal relationship of persistent organic pollutants (POPs) serum concentrations with endogenous sex-related hormones in Hispanic/Latino adults**

**
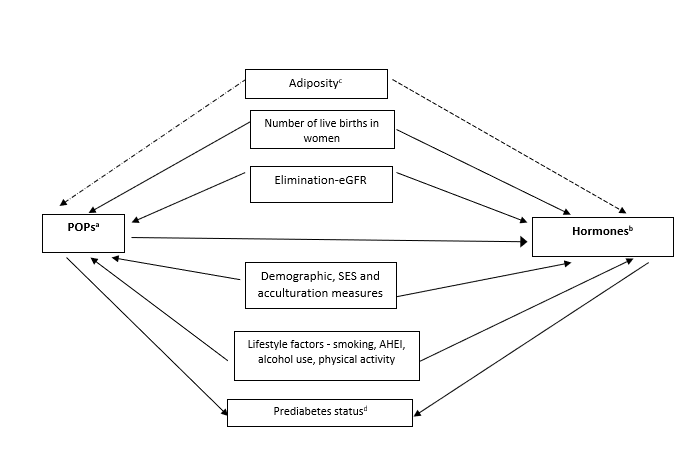
**

^a^ POPs concentrations are lipid adjusted and include polychlorinated biphenyls (PCBs), organochlorine pesticides (OCPs) and brominated flame retardants (BFRs)

^b^ Sex-related hormones include luteinizing hormone (LH), follicle-stimulating hormone (FSH), sex-hormone–binding globulin (SHBG), dehydroepiandrosterone sulfate (DHEAS), estradiol (E2), bioavailable E2; and bioavailable testosterone and testosterone measured in men only.

*A priori* confounders include demographic, socio-economic status (SES) and acculturation measures such as age, HCHS/SOL study sites, education, Hispanic/Latino background, and acculturation score-MESA; number of live births; lifestyle factors such as Alternative healthy eating index-2010, smoking, alcohol use and physical activity; body mass index, waist to hip ratio, and estimated glomerular filtration rate (eGFR)

^c^ Dotted lines represent possible bi-directional relationships which should be taken into consideration when interpreting analysis findings

^d^ Prediabetes status was identified as a potential collider and was not controlled for in models

**Table** **S1. Study selection table for associations of POPs serum concentrations with sex-related hormones in Hispanic/Latino adults**

| **Frequency (n) of POPs congeners with concentrations below the limit of detection (LOD) before exclusions and multiple imputation** | β-HCCH=265; Mirex=1,078; o,p’-DDT=2,098; Oxychlordane=46; p,p’-DDT=1,230; Trans-nonachlor=11; PBB 153=322; PBDE 100=119; PBDE 153=19; PBDE 154=1,564; PBDE 17=2,118; PBDE 183=1,594; PBDE 209=803; PBDE 28=465; PBDE 47=70; PBDE 85=1,427; PBDE 99=476; PCB 105=246; PCB 114=878; PCB 118=2; PCB 138-158=2; PCB 146=14; PCB 156=13; PCB 157=425; PCB 167=293; PCB 170=2; PCB 178=133; PCB 183=67; PCB 187=8; PCB 189=1,120; PCB 194=10; PCB 196-203=7; PCB 199=10; PCB 206=29; PCB 209=123; PCB 28=378; PCB 66=746; PCB 74=5; PCB 99=13 | | |
| --- | --- | --- | --- |
| **Frequency (n) of POPs congeners with concentrations missing or non-reportable before exclusions and multiple imputation** | β-HCCH=34; HCB=49; Mirex=34; o,p’-DDT=51; Oxychlordane=15; p,p’-DDE=17; p,p’-DDT=72; Trans-nonachlor=34; PBB 153=9; PBDE 100=6; PBDE 153=7; PBDE 154=7; PBDE 17=16; PBDE 183=8; PBDE 209=85; PBDE 28=9; PBDE 47=7; PBDE 85=6; PBDE 99=6; PCB 105=49; PCB 114=32; PCB 118=39; PCB 138-158=35; PCB 146=37; PCB 153=9; PCB 156=13; PCB 157=10; PCB 167=22; PCB 170=15; PCB 178=53; PCB 180=55; PCB 183=52; PCB 187=30; PCB 189=20; PCB 194=22; PCB 196-203=24; PCB 199=15; PCB 206=38; PCB 209=13; PCB 28=46; PCB 66=35; PCB 74=36; PCB 99=42 | | |
|  |  | **N Females** | **N Males** |
| **Starting Number** | 2,343 | 1,197 | 1,146 |
| **Pre- or Peri-menopausal Exclusions** |  | 363 | 0 |
| **Medication Exclusions^a^** |  | | |
| Metformin | | 10 | 0 |
| Androgens, Anabolic Steroids, Estrogens, Progestins, | | 41 | 4 |
| Antineoplastic Hormonal Agents, Antiadrenals, Aromatase Inhibitors, Luteinizing Hormone-Releasing Hormone (LHRH) Analogs, Gonadotropin Releasing Hormone (GNRH), LHRH/GNRH Agonists, Hormone Receptor Modulators, Fertility Modulators | | 12 | 0 |
| Vaginal Estrogens and Progestins | | 1 | 0 |
| 5-Alpha Reductase Inhibitors | | 0 | 31 |
| Corticosteroids, Glucocorticoids, Mineralocorticoids | | 11 | 8 |
| Carbamazepine, Phenobarbital, Phenytoin | | 6 | 8 |
| Thyroid and Antithyroid Medications | | 90 | 21 |
| Prolactin Inhibitors, Dopamine Receptor Agonists - D1 And D2, Levodopa, Antiparkinsonian Dopaminergic, Lithium, Amiodarone | | 13 | 10 |
| **Missing sex hormones before multiple imputation** | Females: DHEAS=1 | | |
|  | Males: SHBG=2; E2=2; DHEAS=2; T=3; Bioavailable E2=2; Bioavailable T=3; LH=1; FSH=1 | | |
| **Missing covariates before multiple imputation** | Females: education=1; physical activity level=1; smoking status=1; alcohol use=1; waist to hip ratio=1; AHEI-2010=8; live births=7; acculturation score – MESA=4; eGFR=2 | | |
|  | Males: education=2; BMI=2; physical activity level=5; smoking status=5; alcohol use=2; waist to hip ratio=3; AHEI-2010=11; acculturation score – MESA=6; eGFR=8 | | |
| **Total at baseline examination (V1)** |  | 716 | 1,073 |

^a^ Not all exclusions are mutually exclusive

**Table S2. Polychlorinated biphenyls (PCB) and polybrominated diphenyl ethers (PBDE) groups based on structure, biological and pharmacokinetic function**

| **POPs group** | **Congeners** |
| --- | --- |
| **∑PBDEs** | PBDE 100, 153, 154, 17, 183, 209, 28, 47, 85, 99 |
| **∑PCBs** | PCB 105, 114, 118, 138-158, 156, 153, 156, 157, 167, 170, 178, 180, 183, 187, 189, 194, 196-203, 199, 206, 209, 28, 66, 74, 99 |
| **3-methylcholanthrene inducers-CYP IA inducers/substrates (∑3MC-PCB inducers)** | PCB 66, 74, 105, 118, 156, 167 |
| **∑Dioxin-like PCBs (∑DL-PCBs)** | PCB 105, 114, 118, 156, 157, 167, 189 |
| **∑Non-Dioxin-like PCBs (∑NDL-PCBs)** | PCB 28, 66, 74, 99, 138158, 146, 153, 170, 178, 180, 183, 187, 194, 196-203, 199, 206, 209 |
| **phenobarbital-type inducers-CYP IIB inducers (∑PHB-PCB inducers)** | PCB 99, 153, 180, 183, 187, 194, 196–203, 199, 206 |

**Table S3. Weighted mean (95% CI) levels of sex-related hormones by median POPs concentrations, Hispanic/Latino postmenopausal women (N=716)**

| **POPs concentrations (ng/g Lipid)** | **LH (mIU/mL)** | **FSH (mIU/mL)** | **SHBG (nmol/L)** | **DHEAS (umol/L)** |
| --- | --- | --- | --- | --- |
|  | **AM (95% CI)** | **AM (95% CI)** | **GM (95% CI)** | **GM (95% CI)** |
| **∑PBDEs** |  |  |  |  |
| <= median | 32.3 [30.6,34.1] | 66.7 [62.6,70.8] | 53.1 [49.1,57.5] | 1.83 [1.64,2.04] |
| > median | 33.0 [30.3,35.7] | 64.9 [58.7,71.0] | 56.8 [52.0,62.2] | 1.98 [1.75,2.25] |
| **∑PCBs** |  |  |  |  |
| <= median | 33.4 [31.3,35.6] | 63.9 [60.0,67.9] | 49.0 [45.6,52.7]** | 1.99 [1.73,2.29] |
| > median | 32.1 [30.0,34.2] | 67.2 [61.6,72.7] | 59.3 [54.3,64.8] | 1.84 [1.66,2.03] |
| **∑3MC-PCB inducers** |  |  |  |  |
| <= median | 34.1 [32.0,36.3] | 67.1 [62.9,71.4] | 50.8 [47.0,54.9]** | 2.05 [1.79,2.36] |
| > median | 31.6 [29.5,33.7] | 64.9 [59.7,70.2] | 57.9 [53.2,63.1] | 1.79 [1.62,1.98] |
| **∑DL-PCBs** |  |  |  |  |
| <= median | 34.2 [31.9,36.5] | 66.1 [61.6,70.6] | 49.8 [46.0,53.9]** | 2.07 [1.79,2.40] |
| > median | 31.6 [29.5,33.7] | 65.7 [60.6,70.8] | 58.5 [53.8,63.6] | 1.79 [1.62,1.97] |
| **∑NDL-PCBs** |  |  |  |  |
| <= median | 33.2 [31.1,35.4] | 63.6 [59.6,67.5] | 49.0 [45.6,52.7]** | 1.99 [1.73,2.29] |
| > median | 32.2 [30.1,34.4] | 67.5 [61.9,73.0] | 59.4 [54.3,64.9] | 1.84 [1.66,2.03] |
| **∑PHB-PCB inducers** |  |  |  |  |
| <= median | 33.1 [31.0,35.2] | 63.0 [59.0,66.9] | 49.3 [45.9,53.0]** | 1.97 [1.71,2.27] |
| > median | 32.3 [30.2,34.5] | 67.9 [62.3,73.5] | 59.1 [54.1,64.7] | 1.85 [1.67,2.04] |
| **PBB 153** |  |  |  |  |
| <= median | 33.0 [31.1,34.8] | 66.7 [62.0,71.5] | 51.4 [47.2,56.0]** | 1.97 [1.73,2.23] |
| > median | 32.3 [29.8,34.8] | 64.9 [59.2,70.5] | 58.7 [54.3,63.6] | 1.83 [1.63,2.05] |
| **β-HCCH** |  |  |  |  |
| <= median | 35.6 [33.6,37.7]** | 67.3 [63.3,71.3] | 52.1 [48.4,56.0] | 2.2 [1.95,2.48]** |
| > median | 30.6 [28.5,32.6] | 64.8 [59.2,70.4] | 56.8 [51.9,62.3] | 1.71 [1.54,1.91] |
| **Mirex^a^** |  |  |  |  |
| Non-detected | 34.3 [32.2,36.4] | 67.1 [62.6,71.7] | 52.7 [49.2,56.4] | 1.93 [1.69,2.21] |
| <= Median detected | 28.8 [25.0,32.6]** | 59.9 [50.8,69.1] | 55.6 [46.6,66.4] | 1.89 [1.70,2.09] |
| > Median detected | 32.8 [30.4,35.1] | 68.8 [63.8,73.9] | 58.9 [53.2,65.3] | 1.84 [1.59,2.14] |
| **HCB** |  |  |  |  |
| <= median | 34.5 [32.4,36.7]** | 67.3 [63.3,71.3] | 50.9 [47.1,55.0]** | 2.03 [1.80,2.28] |
| > median | 31.0 [28.8,33.1] | 64.6 [59.0,70.1] | 58.6 [53.5,64.1] | 1.79 [1.60,2.01] |
| **Oxychlordane** |  |  |  |  |
| <= median | 34.7 [32.5,36.9]** | 65.7 [61.7,69.7] | 48.2 [45.0,51.6]** | 2.19 [1.93,2.48]** |
| > median | 31.2 [29.1,33.2] | 66.0 [60.3,71.6] | 60.3 [55.0,66.1] | 1.71 [1.54,1.90] |
| **Trans-nonachlor** |  |  |  |  |
| <= median | 34.1 [31.9,36.4] | 65.5 [61.4,69.6] | 51.2 [47.6,55.0]** | 2.18 [1.92,2.48]** |
| > median | 31.4 [29.3,33.5] | 66.1 [60.3,72.0] | 58 [52.9,63.6] | 1.70 [1.53,1.88] |
| **o,p’-DDT^a^** |  |  |  |  |
| Non-detected | 32.5 [30.8,34.2] | 66.1 [62.0,70.1] | 55.1 [51.6,58.8] | 1.89 [1.74,2.06] |
| Detected | 34.4 [29.3,39.5] | 62.6 [52.0,73.2] | 51.6 [45.6,58.4] | 1.97 [1.50,2.58] |
| **p,p’-DDE** |  |  |  |  |
| <= median | 33.7 [31.7,35.8] | 68.8 [64.6,72.9] | 52.7 [48.2,57.6] | 1.89 [1.70,2.11] |
| > median | 31.6 [29.1,34.1] | 63.1 [57.4,68.8] | 56.9 [52.6,61.6] | 1.90 [1.68,2.15] |
| **p,p’-DDT^a^** |  |  |  |  |
| Non-detected | 34.7 [32.7,36.7] | 68.3 [64.3,72.4] | 54.1 [50.0,58.5] | 1.98 [1.78,2.21] |
| <= Median detected | 32.7 [30.0,35.5] | 69.9 [63.7,76.2] | 57.8 [52.5,63.7] | 1.91 [1.66,2.20] |
| > Median detected | 29.4 [26.1,32.6]** | 58.5 [50.0,67.0]** | 53.5 [46.3,61.8] | 1.77 [1.48,2.12] |

AM: Arithmetic mean; GM: Geometric mean

∑PBDEs — PBDE 100, 153, 154, 17, 183, 209, 28, 47, 85, 99

∑PCBs — PCB 105, 114, 118, 138-158, 156, 153, 156, 157, 167, 170, 178, 180, 183, 187, 189, 194, 196-203, 199, 206, 209, 28, 66, 74, 99

∑3-methylcholanthrene (3MC) inducers: CYP IA inducers/substrates — PCB 66, 74, 105, 118, 156, 167

∑Dioxin-like PCBs — PCB 105, 114, 118, 156, 157, 167, 189

∑Non-Dioxin-like PCBs — PCB 28, 66, 74, 99, 138158, 146, 153, 170, 178, 180, 183, 187, 194, 196-203, 199, 206, 209

∑Phenobarbital-type (PB) inducers: CYP IIB inducers – PCB 99, 153, 180, 183, 187, 194, 196–203, 199, 206

^a^ o,p’-DDT: non-detected (n=666), detected (n=50); Mirex: non-detected (n=381), <= median detected (n=168), > median detected (n=167); p,p’-DDT: non-detected (n=342), <= median detected (n=188), > median detected (n=186). Only 7% of samples had o,p’-DDT values above LOD and this should be taken into consideration when interpreting findings

Overall p value for mirex: LH (0.147), FSH (0.960), SHBG (0.081), DHEAS (0.655)

Overall p value for p,p’-DDT: LH (0.007), FSH (0.053), SHBG (0.976), DHEAS (0.298)

** P<0.05

**Table S4. Weighted mean (95% CI) levels of sex-related hormones by median POPs concentrations, Hispanic/Latino men (N=1,073)**

| **POPs concentrations (ng/g Lipid)** | **LH (mIU/mL)** | **FSH (mIU/mL)** | **SHBG (nmol/L)** | **E2 (pmol/L)** | **Bio E2 (pmol/L)** | **DHEAS (umol/L)** | **T (ng/dL)** | **Bio T (ng/dL)** |
| --- | --- | --- | --- | --- | --- | --- | --- | --- |
|  | **GM (95% CI)** | **GM (95% CI)** | **GM (95% CI)** | **AM (95% CI)** | **AM (95% CI)** | **AM (95% CI)** | **AM (95% CI)** | **AM (95% CI)** |
| **∑PBDEs** |  |  |  |  |  |  |  |  |
| <= median | 5.83 [5.41,6.29] | 5.71 [5.19,6.29] | 45.3 [42.3,48.6] | 93.5 [87.9,99.1] | 60.7 [57.0,64.4] | 4.49 [4.14,4.83] | 435.3 [409.6,461.0] | 161.3 [154.7,168.0] |
| > median | 5.54 [5.23,5.86] | 5.85 [5.49,6.23] | 45.9 [42.9,49.1] | 90.4 [85.4,95.3] | 58.3 [55.1,61.6] | 4.40 [4.05,4.75] | 425.0 [406.6,443.3] | 158.1 [151.8,164.5] |
| **∑PCBs** |  |  |  |  |  |  |  |  |
| <= median | 5.42 [5.10,5.76] | 5.19 [4.81,5.59]** | 40.6 [38.1,43.3]** | 94.9 [89.6,100.1] | 64.5 [60.6,68.4]** | 4.94 [4.63,5.24]** | 422.6 [401.5,443.7] | 168.7 [162.7,174.7]** |
| > median | 5.90 [5.50,6.33] | 6.27 [5.74,6.85] | 49.9 [47.0,52.9] | 89.8 [84.4,95.2] | 55.8 [52.7,58.8] | 4.06 [3.74,4.39] | 436.2 [412.9,459.6] | 153.0 [146.4,159.5] |
| **∑3MC-PCB inducers** |  |  |  |  |  |  |  |  |
| <= median | 5.47 [5.13,5.85] | 5.22 [4.85,5.62]** | 43.5 [40.5,46.6] | 94.1 [88.4,99.8] | 62.4 [58.3,66.5] | 4.76 [4.45,5.07]** | 434.5 [411.7,457.3] | 165.8 [159.6,172.1]** |
| > median | 5.86 [5.47,6.28] | 6.26 [5.72,6.85] | 47.4 [44.6,50.4] | 90.3 [85.2,95.4] | 57.3 [54.4,60.3] | 4.19 [3.85,4.54] | 426.9 [403.6,450.3] | 155.0 [148.7,161.3] |
| **∑DL-PCBs** |  |  |  |  |  |  |  |  |
| <= median | 5.39 [5.06,5.75] | 5.14 [4.77,5.54]** | 42.9 [40.0,46.0]** | 94.0 [88.2,99.8] | 62.7 [58.5,66.9]** | 4.80 [4.48,5.12]** | 430.2 [407.9,452.5] | 166.0 [159.7,172.2]** |
| > median | 5.93 [5.53,6.36] | 6.32 [5.78,6.90] | 47.8 [45.0,50.8] | 90.4 [85.4,95.5] | 57.2 [54.3,60.0] | 4.17 [3.84,4.50] | 430.4 [406.8,454.0] | 155.0 [148.8,161.3] |
| **∑NDL-PCBs** |  |  |  |  |  |  |  |  |
| <= median | 5.44 [5.12,5.77] | 5.22 [4.84,5.62]** | 40.4 [37.9,43.0]** | 95.1 [89.9,100.4] | 64.8 [60.9,68.7]** | 4.95 [4.65,5.26]** | 420.5 [399.6,441.4] | 168.4 [162.4,174.4]** |
| > median | 5.89 [5.48,6.32] | 6.25 [5.72,6.83] | 50.1 [47.2,53.1] | 89.6 [84.2,95.0] | 55.5 [52.5,58.6] | 4.05 [3.73,4.37] | 437.9 [414.4,461.3] | 153.2 [146.6,159.7] |
| **∑PHB-PCB inducers** |  |  |  |  |  |  |  |  |
| <= median | 5.47 [5.15,5.81] | 5.29 [4.92,5.70]** | 40.4 [37.8,43.2]** | 97.9 [92.3,103.5]** | 66.2 [62.5,70.0]** | 4.94 [4.64,5.25]** | 423.7 [403.6,443.9] | 168.9 [163.8,174.0]** |
| > median | 5.86 [5.45,6.30] | 6.17 [5.64,6.75] | 49.9 [47.1,52.9] | 87.6 [82.6,92.6] | 54.6 [51.6,57.6] | 4.07 [3.74,4.39] | 435.2 [411.0,459.5] | 153.0 [145.9,160.0] |
| **PBB 153** |  |  |  |  |  |  |  |  |
| <= median | 5.77 [5.44,6.13] | 5.82 [5.46,6.21] | 43.4 [40.9,46.0]** | 91.8 [86.3,97.2] | 61.0 [57.2,64.9] | 4.52 [4.12,4.92] | 410.9 [390.2,431.6]** | 158.0 [151.2,164.8] |
| > median | 5.59 [5.18,6.03] | 5.73 [5.17,6.34] | 48.3 [44.8,52.1] | 92.2 [86.7,97.8] | 57.9 [54.8,61.0] | 4.36 [4.08,4.64] | 452.5 [427.7,477.4] | 161.9 [156.3,167.4] |
| **β-HCCH** |  |  |  |  |  |  |  |  |
| <= median | 5.80 [5.36,6.28] | 5.59 [5.07,6.17] | 49.0 [46.0,52.1]** | 96.8 [91.2,102.3]** | 61.3 [58.1,64.5] | 4.77 [4.44,5.10]** | 472.2 [444.4,500.1]** | 169.8 [163.0,176.6]** |
| > median | 5.60 [5.25,5.97] | 5.93 [5.49,6.41] | 43.1 [40.4,46.0] | 88.2 [83.3,93.1] | 58.1 [54.7,61.6] | 4.18 [3.86,4.51] | 396.7 [382.0,411.5] | 151.8 [146.3,157.3] |
| **Mirex^a^** |  |  |  |  |  |  |  |  |
| Non-detected | 5.62 [5.25,6.01] | 5.49 [5.00,6.04] | 43.6 [40.4,47.0] | 96.7 [89.4,104.0] | 63.7 [59.2,68.1] | 4.35 [4.03,4.67] | 418.8 [397.4,440.3] | 160.5 [155.2,165.9] |
| <= Median detected | 5.44 [5.08,5.84] | 5.57 [5.12,6.06] | 41.7 [39.1,44.6] | 89.8 [83.9,95.6] | 60.2 [55.9,64.5] | 4.81 [4.25,5.36] | 415.6 [387.8,443.5] | 162.0 [154.0,170.0] |
| > Median detected | 6.02 [5.42,6.68] | 6.35 [5.60,7.20] | 52.3 [47.9,57.1]** | 88.3 [81.5,95.1] | 54.0 [50.2,57.9]** | 4.22 [3.86,4.57] | 457.8 [423.3,492.3]** | 156.9 [147.3,166.5] |
| **HCB** |  |  |  |  |  |  |  |  |
| <= median | 5.98 [5.58,6.42] | 5.89 [5.37,6.45] | 47.1 [44.0,50.4] | 96.3 [90.1,102.4]** | 61.5 [57.8,65.2] | 4.38 [4.05,4.71] | 435.0 [409.0,460.9] | 157.1 [150.2,164.2] |
| > median | 5.41 [5.03,5.81] | 5.67 [5.23,6.15] | 44.2 [41.6,46.9] | 87.7 [83.2,92.2] | 57.6 [54.5,60.7] | 4.50 [4.15,4.85] | 425.6 [405.6,445.6] | 162.4 [156.8,168.1] |
| **Oxychlordane** |  |  |  |  |  |  |  |  |
| <= median | 5.56 [5.25,5.89] | 5.38 [5.00,5.78]** | 42.0 [39.4,44.9]** | 94.6 [90.5,98.7] | 63.3 [60.5,66.2]** | 4.88 [4.58,5.18]** | 439.6 [417.0,462.2] | 170.8 [165.9,175.8]** |
| > median | 5.80 [5.38,6.26] | 6.15 [5.59,6.76] | 48.9 [46.0,52.1] | 89.7 [83.8,95.6] | 56.2 [52.6,59.9] | 4.06 [3.72,4.40] | 422.2 [399.4,445.0] | 150.2 [143.3,157.0] |
| **Trans-nonachlor** |  |  |  |  |  |  |  |  |
| <= median | 5.67 [5.34,6.03] | 5.4 [5.02,5.81]** | 42.7 [40.2,45.3]** | 94.2 [89.3,99.2] | 63.0 [59.6,66.5]** | 4.91 [4.61,5.22]** | 435.6 [411.7,459.5] | 168.2 [162.0,174.5]** |
| > median | 5.70 [5.32,6.10] | 6.09 [5.56,6.66] | 48.0 [44.9,51.4] | 90.2 [85.0,95.5] | 56.9 [53.6,60.1] | 4.07 [3.75,4.40] | 426.2 [404.1,448.3] | 153.2 [146.9,159.5] |
| **o,p’-DDT^a^** |  |  |  |  |  |  |  |  |
| Non-detected | 5.73 [5.46,6.01] | 5.79 [5.46,6.15] | 45.4 [43.3,47.6] | 93.0 [89.1,96.8] | 60.3 [57.9,62.7] | 4.50 [4.25,4.74] | 437.1 [420.7,453.6]** | 162.4 [158.6,166.3]** |
| Detected | 5.16 [4.05,6.59] | 5.58 [4.09,7.63] | 48.0 [38.8,59.5] | 79.5 [63.9,95.2] | 50.6 [39.1,62.0] | 3.73 [2.98,4.48] | 342.9 [286.9,398.9] | 125.9 [100.2,151.6] |
| **p,p’-DDE** |  |  |  |  |  |  |  |  |
| <= median | 5.87 [5.46,6.32] | 5.56 [5.05,6.12] | 47.2 [43.8,50.8] | 95.3 [90.1,100.6] | 61.1 [57.8,64.5] | 4.86 [4.48,5.24]** | 456.2 [428.8,483.7]** | 165.3 [158.9,171.7]** |
| > median | 5.52 [5.18,5.88] | 5.99 [5.54,6.47] | 44.2 [41.7,46.8] | 88.8 [83.5,94.2] | 58.1 [54.5,61.6] | 4.05 [3.77,4.32] | 405.9 [388.3,423.5] | 154.6 [148.2,161.0] |
| **p,p’-DDT^a^** |  |  |  |  |  |  |  |  |
| Non-detected | 5.62 [5.30,5.95] | 5.42 [5.10,5.76] | 46.3 [43.6,49.2] | 98.8 [93.7,103.9] | 64.1 [60.6,67.6] | 4.43 [4.10,4.76] | 454.7 [437.3,472.0] | 168.1 [163.5,172.8] |
| <= Median detected | 5.81 [5.11,6.61] | 6.18 [5.17,7.38] | 43.6 [38.8,49.0] | 81.6 [72.8,90.3]** | 52.7 [48.2,57.2]** | 4.80 [4.23,5.38] | 403.9 [356.2,451.6]** | 150.9 [140.6,161.2]** |
| > Median detected | 5.75 [5.21,6.33] | 6.30 [5.54,7.15]** | 45.8 [41.7,50.3] | 85.8 [79.0,92.7]** | 55.4 [50.8,59.9]** | 4.14 [3.71,4.56] | 398.1 [365.1,431.2]** | 148.7 [136.9,160.5]** |

AM: Arithmetic mean; GM: Geometric mean

∑PBDEs — PBDE 100, 153, 154, 17, 183, 209, 28, 47, 85, 99

∑PCBs — PCB 105, 114, 118, 138-158, 156, 153, 156, 157, 167, 170, 178, 180, 183, 187, 189, 194, 196-203, 199, 206, 209, 28, 66, 74, 99

∑3-methylcholanthrene (3MC) inducers: CYP IA inducers/substrates — PCB 66, 74, 105, 118, 156, 167

∑Dioxin-like PCBs — PCB 105, 114, 118, 156, 157, 167, 189

∑Non-Dioxin-like PCBs — PCB 28, 66, 74, 99, 138158, 146, 153, 170, 178, 180, 183, 187, 194, 196-203, 199, 206, 209

∑Phenobarbital-type (PB) inducers: CYP IIB inducers – PCB 99, 153, 180, 183, 187, 194, 196–203, 199, 206

^a^ o,p’-DDT: non-detected (n=1,010), detected (n=63); Mirex: non-detected (n=413), <= median detected (n=334), > median detected (n=326); p,p’-DDT: non-detected (n=615), <= median detected (n=219), > median detected (n=235). Only 7% of samples had o,p’-DDT values above LOD and this should be taken into consideration when interpreting findings

Overall p value for mirex: LH (0.284), FSH (0.07), SHBG (0.003), E2 (0.116), Bio E2 (0.003), DHEAS (0.678), T (0.065), Bio T (0.531)

Overall p value for p,p’-DDT: LH (0.636), FSH (0.021), SHBG (0.693), E2 (<0.0001), Bio E2 (<0.0001), DHEAS (0.524), T (0.001), Bio T (0.001)

** P<0.05

**Table S5. Weighted mean (95% CI) levels of sex-related hormones by demographic, clinical and lifestyle characteristics, Hispanic/Latino postmenopausal women (N=716)**

| **Characteristics** | **LH (mIU/mL)** | **FSH (mIU/mL)** | **SHBG (nmol/L)** | **DHEAS (umol/L)** |
| --- | --- | --- | --- | --- |
|  | **AM (95% CI)** | **AM (95% CI)** | **GM (95% CI)** | **GM (95% CI)** |
| **Age** |  |  |  |  |
| Age 45-54 | 34.9 [31.8,37.9]** | 64.9 [59.9,70.0] | 50.4 [46.2,54.9] | 2.59 [2.28,2.93]** |
| Age 55-64 | 34.5 [33.2,35.9] | 69.4 [64.9,73.8] | 55.0 [50.5,59.8] | 1.75 [1.54,1.99] |
| Age 65+ | 27.7 [24.4,30.9] | 61.8 [52.8,70.8] | 59.7 [51.2,69.6] | 1.54 [1.33,1.78] |
| **Hispanic/Latino background** |  |  |  |  |
| Dominican | 34.6 [31.1,38.0]** | 66.9 [58.9,74.9] | 55.5 [48.2,63.8] | 1.98 [1.75,2.25] |
| Central American | 34.0 [28.9,39.1] | 64.2 [55.4,73.1] | 43.8 [39.0,49.2]** | 2.06 [1.65,2.56] |
| Cuban | 33.0 [29.2,36.8] | 72.1 [61.5,82.7]** | 57.5 [48.9,67.8] | 1.97 [1.70,2.29] |
| Mexican (Reference) | 30.4 [28.2,32.5] | 59.6 [55.2,64.0] | 52.8 [48.9,57.0] | 1.75 [1.46,2.09] |
| Puerto Rican | 31.5 [28.9,34.2] | 63.1 [56.1,70.1] | 58.2 [51.4,65.8] | 1.98 [1.73,2.27] |
| South American | 36.8 [32.4,41.1]** | 68.5 [61.1,76.0]** | 51.5 [41.9,63.3] | 1.91 [1.55,2.34] |
| More than one or other heritage | 43.4 [28.4,58.4] | 83.8 [57.0,110.6] | 63.1 [42.5,93.5] | 1.85 [0.83,4.14] |
| **Acculturation score - MESA** |  |  |  |  |
| <= median | 33.6 [32.0,35.1] | 67.9 [64.3,71.5] | 52.3 [49.0,55.9]** | 1.93 [1.75,2.13] |
| > median | 30.3 [26.7,33.8] | 60.6 [52.2,69.0] | 61.8 [55.0,69.4] | 1.82 [1.54,2.15] |
| **Educational attainment** |  |  |  |  |
| Less than high school | 30.9 [29.1,32.8] | 62.7 [58.1,67.2] | 54.9 [50.6,59.6] | 1.70 [1.49,1.94] |
| High school diploma or GED | 34.1 [30.9,37.3] | 69.4 [62.7,76.1] | 62.4 [58.3,66.9] | 2.17 [1.89,2.50] |
| Greater than high school diploma or GED | 33.7 [30.8,36.6] | 67.4 [60.5,74.3] | 52.1 [46.6,58.2] | 1.99 [1.75,2.27] |
| **Body mass index** |  |  |  |  |
| Under or normal weight (BMI < 25) | 37.9 [35.0,40.8]** | 79.4 [70.6,88.2]** | 70.5 [61.0,81.4]** | 2.18 [1.94,2.45] |
| Overweight (25 ≤ BMI < 30) | 35.1 [33.0,37.2] | 73.0 [68.5,77.5] | 58.5 [54.4,63.0] | 1.89 [1.70,2.11] |
| Obese (BMI ≥ 30) | 27.8 [25.4,30.2] | 52.5 [48.2,56.9] | 45.7 [40.5,51.6] | 1.78 [1.52,2.09] |
| **High WHR (≥ 0.85)** |  |  |  |  |
| Yes | 34.6 [31.7,37.4] | 71.0 [64.9,77.1] | 62.6 [56.3,69.6]** | 2.09 [1.81,2.40] |
| No | 32.1 [30.3,33.9] | 64.4 [60.3,68.6] | 52.8 [49.2,56.8] | 1.85 [1.68,2.04] |
| **Physical activity level** |  |  |  |  |
| High | 36.5 [25.1,47.9] | 70.2 [52.9,87.5] | 57.4 [45.0,73.3] | 2.68 [1.86,3.87] |
| Moderate | 33.5 [30.6,36.4] | 65.5 [59.5,71.6] | 57.0 [52.2,62.2] | 1.95 [1.75,2.17] |
| Low | 31.7 [29.8,33.5] | 65.7 [61.2,70.3] | 53.0 [48.8,57.5] | 1.81 [1.61,2.04] |
| **Smoking status** |  |  |  |  |
| Never (Reference) | 31.9 [29.8,33.9] | 64.5 [59.6,69.4] | 53.7 [49.5,58.3] | 1.78 [1.59,1.98] |
| Former | 33.7 [30.9,36.6] | 66.5 [60.3,72.6] | 56.1 [49.6,63.4] | 1.96 [1.69,2.26] |
| Current | 34.7 [31.9,37.5] | 70.9 [64.0,77.9] | 58.5 [52.4,65.4] | 2.43 [2.06,2.86]** |
| **Alcohol use** |  |  |  |  |
| None | 32.4 [30.3,34.5] | 65.3 [60.6,70.0] | 53.4 [49.3,57.8] | 1.74 [1.56,1.94]** |
| Low | 33.2 [31.0,35.5] | 67.3 [62.1,72.5] | 58.0 [53.6,62.8] | 2.25 [2.03,2.49] |
| High | 30.7 [26.4,35.0] | 55.7 [46.3,65.0] | 51.1 [34.9,74.9] | 1.95 [1.24,3.08] |
| **eGFR** |  |  |  |  |
| <= median | 32.6 [30.3,34.9] | 66.0 [60.7,71.4] | 55.5 [51.3,60.0] | 1.69 [1.51,1.90]** |
| > median | 32.7 [30.8,34.6] | 65.6 [61.1,70.1] | 53.9 [49.0,59.3] | 2.23 [1.98,2.50] |
| **Diet quality score (AHEI-2010)** |  |  |  |  |
| <= median | 33.2 [30.8,35.6] | 66.0 [60.5,71.5] | 53.3 [48.6,58.4] | 2.06 [1.87,2.27]** |
| > median | 32.0 [30.0,34.0] | 65.7 [60.9,70.5] | 56.6 [52.5,61.1] | 1.74 [1.51,1.99] |
| **Number of prior live births (females only)** |  |  |  |  |
| 0-1 | 35.0 [31.4,38.6] | 72.2 [64.8,79.6] | 55.6 [48.7,63.4] | 2.23 [1.89,2.64] |
| 2 | 32.0 [28.9,35.0] | 64.9 [57.2,72.5] | 53.8 [47.3,61.1] | 1.88 [1.69,2.09] |
| 3-4 | 34.4 [32.2,36.6] | 68.6 [63.7,73.6] | 56.0 [51.8,60.6] | 1.97 [1.73,2.24] |
| 5+ | 27.2 [24.3,30.1]** | 53.6 [47.6,59.7]** | 53.5 [46.3,61.8] | 1.46 [1.13,1.89]** |
| **Recruitment center** |  |  |  |  |
| Bronx | 32.5 [29.6,35.4] | 64.7 [58.9,70.5] | 54.8 [49.5,60.7] | 1.96 [1.76,2.19] |
| Chicago (Reference) | 29.6 [27.5,31.7] | 57.4 [52.5,62.3] | 56.2 [50.5,62.6] | 1.77 [1.53,2.06] |
| Miami | 33.7 [30.5,36.9]** | 70.3 [62.4,78.3]** | 54.5 [47.9,61.9] | 1.94 [1.69,2.22] |
| San Diego | 32.6 [30.0,35.2] | 64.0 [57.8,70.2] | 54.8 [50.5,59.6] | 1.81 [1.41,2.33] |

AM: Arithmetic mean; GM: Geometric mean

Overall p value for Hispanic/Latino background: LH (0.748), FSH (0.878), SHBG (0.411), DHEAS (0.601)

Overall p value for smoking status: LH (0.092), FSH (0.139), SHBG (0.216), DHEAS (0.003)

Overall p value for number of live births: LH (0.046), FSH (0.009), SHBG (0.932), DHEAS (0.026)

Overall p value for recruitment center: LH (0.622), FSH (0.512), SHBG (0.935), DHEAS (0.663)

Differences in mean hormone levels were evaluated for ordinal variables using overall tests. We conducted pairwise contrasts for nominal variables regardless of whether overall tests were significant or non-significant. When overall tests are not significant, pairwise contrasts should be interpreted with caution since post-hoc adjustment was not conducted.

** P<0.05

**Table S6. Weighted mean (95% CI) levels of sex-related hormones by demographic, clinical and lifestyle characteristics, Hispanic/Latino men (N=1,073)**

| **Characteristics** | **LH (mIU/mL)** | **FSH (mIU/mL)** | **SHBG (nmol/L)** | **E2 (pmol/L)** | **Bio E2 (pmol/L)** | **DHEAS (umol/L)** | **T (ng/dL)** | **Bio T (ng/dL)** |
| --- | --- | --- | --- | --- | --- | --- | --- | --- |
|  | **GM (95% CI)** | **GM (95% CI)** | **GM (95% CI)** | **AM (95% CI)** | **AM (95% CI)** | **AM (95% CI)** | **AM (95% CI)** | **AM (95% CI)** |
| **Age** |  |  |  |  |  |  |  |  |
| Age 45-54 | 5.36 [5.07,5.66]** | 5.15 [4.85,5.46]** | 40.1 [37.7,42.6]** | 92.2 [88.3,96.0] | 62.6 [59.9,65.3]** | 5.43 [5.11,5.74]** | 429.3 [410.2,448.5] | 172.9 [168.2,177.7]** |
| Age 55-64 | 5.68 [5.27,6.12] | 6.03 [5.48,6.64] | 48.0 [44.8,51.4] | 91.7 [84.5,98.9] | 58.5 [53.5,63.6] | 3.91 [3.60,4.23] | 421.5 [390.8,452.3] | 149.6 [141.2,158.0] |
| Age 65+ | 6.74 [5.70,7.96] | 7.41 [5.93,9.25] | 59.7 [53.0,67.3] | 92 [79.8,104.3] | 52.8 [46.3,59.3] | 2.62 [2.31,2.94] | 448.6 [402.7,494.4] | 141.0 [128.7,153.3] |
| **Hispanic/Latino background** |  |  |  |  |  |  |  |  |
| Dominican | 6.19 [5.52,6.93]** | 5.29 [4.34,6.44] | 42.0 [37.8,46.8] | 97.1 [85.0,109.3]** | 65.4 [56.0,74.8] | 4.30 [3.67,4.94] | 419.9 [386.4,453.4] | 166.4 [153.3,179.5] |
| Central American | 5.96 [5.07,6.99] | 6.20 [5.32,7.22] | 43.6 [37.4,50.9] | 98.2 [83.9,112.5] | 65.4 [55.2,75.6] | 4.48 [3.94,5.02] | 452.7 [373.1,532.4] | 171.6 [153.5,189.7] |
| Cuban | 5.83 [5.36,6.34] | 6.08 [5.52,6.71] | 46.1 [42.5,50.0] | 90.3 [82.8,97.9] | 58.2 [53.6,62.8] | 4.31 [3.78,4.84] | 416.9 [391.3,442.5] | 153.0 [143.7,162.2] |
| Mexican (Reference) | 5.20 [4.87,5.54]** | 5.36 [4.92,5.85] | 41.4 [38.3,44.8] | 83.7 [80.0,87.4] | 55.8 [52.7,58.9] | 4.45 [4.15,4.74] | 413.5 [394.2,432.8] | 162.4 [157.7,167.1] |
| Puerto Rican | 6.33 [5.30,7.54]** | 6.32 [5.04,7.94] | 58.3 [51.8,65.7]** | 97.9 [86.5,109.2]** | 57.6 [51.4,63.7] | 4.62 [3.81,5.43] | 485.3 [428.2,542.5]** | 156.0 [141.3,170.7] |
| South American | 5.07 [4.29,5.99] | 5.31 [4.60,6.13] | 39.2 [34.7,44.4] | 104.1 [82.2,126.0]** | 71.3 [55.2,87.4]** | 4.81 [4.35,5.26] | 403.8 [367.6,440.1] | 164.7 [154.1,175.3] |
| More than one or other heritage | 5.68 [4.42,7.30] | 5.88 [4.28,8.08] | 52.5 [43.3,63.7]** | 108.2 [88.8,127.6]** | 68.2 [57.4,79.1]** | 4.51 [3.60,5.42] | 460.9 [361.1,560.7] | 162.7 [137.5,187.9] |
| **Acculturation score - MESA** |  |  |  |  |  |  |  |  |
| <= median | 5.72 [5.44,6.02] | 5.78 [5.43,6.15] | 43.8 [41.4,46.3]** | 91.2 [86.7,95.7] | 60.0 [57.0,63.0] | 4.44 [4.16,4.72] | 423.1 [405.3,441.0] | 160.6 [155.4,165.7] |
| > median | 5.60 [5.03,6.24] | 5.78 [5.03,6.64] | 50.6 [46.7,54.8] | 94.0 [87.3,100.7] | 58.5 [54.9,62.1] | 4.45 [3.97,4.93] | 448.4 [413.9,482.8] | 157.8 [148.9,166.7] |
| **Educational attainment** |  |  |  |  |  |  |  |  |
| Less than high school | 5.78 [5.31,6.30] | 6.15 [5.47,6.91] | 48.4 [44.6,52.6] | 88.9 [82.9,95.0] | 56.0 [52.2,59.8]** | 4.03 [3.64,4.42] | 438.3 [412.7,464.0] | 155.9 [150.1,161.7] |
| High school diploma or GED | 5.7 [5.22,6.21] | 5.80 [5.26,6.39] | 44.6 [39.9,49.8] | 93.7 [84.8,102.7] | 60.3 [54.9,65.8] | 4.46 [3.87,5.04] | 420.8 [392.0,449.6] | 158.7 [147.1,170.3] |
| Greater than high school diploma or GED | 5.61 [5.21,6.03] | 5.47 [5.01,5.97] | 43.8 [40.9,47.0] | 93.7 [88.3,99.1] | 62.2 [58.4,65.9] | 4.78 [4.42,5.15] | 428.4 [400.8,456.0] | 163.6 [156.1,171.2] |
| **Body mass index** |  |  |  |  |  |  |  |  |
| Under or normal weight(BMI < 25) | 6.7 [5.96,7.52]** | 6.23 [5.28,7.36] | 59.1 [53.7,65.0]** | 90.4 [81.3,99.5] | 53.3 [47.8,58.9]** | 4.10 [3.67,4.54]** | 524.6 [486.2,563.1]** | 167.7 [158.5,177.0]** |
| Overweight (25 ≤ BMI < 30) | 5.49 [5.18,5.83] | 5.65 [5.26,6.07] | 44.9 [41.9,48.2] | 90.8 [85.8,95.7] | 59.4 [55.7,63.0] | 4.35 [4.05,4.66] | 436.0 [416.5,455.6] | 165.9 [159.4,172.4] |
| Obese (BMI ≥ 30) | 5.28 [4.87,5.73] | 5.64 [5.12,6.21] | 38.0 [35.5,40.8] | 95.1 [88.2,102.1] | 64.8 [60.8,68.8] | 4.85 [4.30,5.40] | 346.7 [326.3,367.1] | 143.8 [136.3,151.3] |
| **High WHR (≥ 0.90)** |  |  |  |  |  |  |  |  |
| Yes | 6.15 [5.22,7.24] | 5.89 [4.62,7.52] | 55.7 [48.1,64.5]** | 93.8 [80.9,106.6] | 56.4 [48.8,64.0] | 4.55 [3.95,5.16] | 509.0 [463.2,554.7]** | 168.8 [156.8,180.8] |
| No | 5.61 [5.36,5.88] | 5.76 [5.45,6.09] | 44.1 [41.9,46.4] | 91.7 [87.6,95.7] | 60.1 [57.4,62.8] | 4.42 [4.17,4.68] | 417.2 [400.3,434.2] | 158.3 [153.4,163.2] |
| **Physical activity level** |  |  |  |  |  |  |  |  |
| High | 5.62 [5.06,6.24] | 5.78 [5.09,6.57] | 42.0 [37.4,47.2] | 91.3 [83.7,98.8] | 60.3 [55.5,65.1] | 4.99 [4.30,5.68] | 417.5 [381.8,453.3] | 160.7 [151.4,170.0] |
| Moderate | 5.93 [5.51,6.39] | 5.83 [5.25,6.48] | 46.1 [43.3,49.2] | 92.9 [87.9,98.0] | 60.1 [57.0,63.3] | 4.45 [4.14,4.76] | 447.1 [423.7,470.5] | 164.8 [159.1,170.5] |
| Low | 5.45 [5.09,5.84] | 5.71 [5.30,6.16] | 46.4 [42.9,50.2] | 91.2 [84.5,97.8] | 58.6 [54.1,63.2] | 4.23 [3.80,4.66] | 416.4 [390.9,441.9] | 153.9 [145.5,162.2] |
| **Smoking status** |  |  |  |  |  |  |  |  |
| Never (Reference) | 5.76 [5.33,6.22] | 5.82 [5.21,6.51] | 43.9 [40.7,47.3] | 93.6 [88.4,98.9] | 61.5 [58.5,64.5] | 4.37 [3.98,4.75] | 443.1 [415.8,470.5] | 168.0 [162.2,173.9] |
| Former | 5.50 [5.13,5.90] | 5.85 [5.35,6.39] | 43.2 [39.4,47.3] | 89.6 [83.1,96.1] | 58.7 [54.2,63.2] | 4.33 [3.88,4.78] | 399.8 [378.1,421.4]** | 153.2 [145.8,160.5]** |
| Current | 5.84 [5.31,6.42] | 5.62 [5.08,6.22] | 51.8 [47.9,56.0]** | 92.7 [84.1,101.3] | 57.8 [52.2,63.5] | 4.70 [4.28,5.11] | 451.7 [419.6,483.7] | 156.5 [146.6,166.3]** |
| **Alcohol use** |  |  |  |  |  |  |  |  |
| None | 5.61 [5.17,6.09] | 5.80 [5.23,6.44] | 49.1 [45.1,53.5]** | 90.4 [84.2,96.5] | 56.7 [52.9,60.4]** | 4.22 [3.82,4.62] | 435.5 [405.7,465.3] | 154.4 [146.2,162.7] |
| Low | 5.78 [5.48,6.10] | 5.63 [5.26,6.03] | 42.8 [40.7,45.2] | 91.7 [87.1,96.3] | 60.8 [57.8,63.9] | 4.60 [4.29,4.91] | 428.4 [409.1,447.7] | 164.7 [159.8,169.7] |
| High | 5.52 [4.64,6.55] | 6.65 [5.33,8.29] | 44.0 [39.7,48.7] | 104.0 [89.7,118.3] | 68.6 [58.0,79.3] | 4.74 [4.02,5.47] | 410.9 [378.4,443.5] | 159.2 [145.9,172.4] |
| **eGFR** |  |  |  |  |  |  |  |  |
| <= median | 6.15 [5.75,6.59]** | 6.17 [5.62,6.78]** | 49.2 [46.0,52.5]** | 94.1 [88.3,99.9] | 59.1 [55.6,62.5] | 4.12 [3.74,4.49]** | 429.7 [406.0,453.4] | 151.5 [144.8,158.2]** |
| > median | 5.22 [4.94,5.51] | 5.37 [5.02,5.75] | 42.0 [39.3,44.9] | 89.7 [85.1,94.3] | 60.1 [56.8,63.4] | 4.80 [4.52,5.09] | 431.0 [408.7,453.2] | 168.9 [163.8,173.9] |
| **Diet quality score (AHEI-2010)** |  |  |  |  |  |  |  |  |
| <= median | 5.86 [5.51,6.24] | 5.89 [5.50,6.31] | 47.1 [44.7,49.5] | 93.1 [87.4,98.9] | 60.1 [56.5,63.7] | 4.54 [4.19,4.88] | 427.1 [407.2,446.9] | 156.6 [149.9,163.3] |
| > median | 5.50 [5.13,5.89] | 5.65 [5.11,6.25] | 44.0 [40.6,47.7] | 90.7 [85.6,95.7] | 59.0 [55.8,62.1] | 4.34 [4.02,4.65] | 434.0 [408.1,459.8] | 163.4 [158.3,168.6] |
| **Recruitment center** |  |  |  |  |  |  |  |  |
| Bronx | 6.24 [5.58,6.97] | 5.83 [4.97,6.84] | 51.3 [46.9,56.2]** | 99.1 [90.5,107.7]** | 61.4 [56.6,66.1] | 4.42 [3.89,4.96] | 464.5 [421.6,507.5] | 162.4 [152.0,172.8] |
| Chicago (Reference) | 5.84 [5.28,6.47] | 5.49 [4.88,6.16] | 44.7 [40.9,48.8] | 88.7 [83.7,93.8] | 57.8 [54.3,61.4] | 4.66 [4.29,5.03] | 441.7 [414.7,468.7] | 166.8 [160.3,173.4] |
| Miami | 5.86 [5.42,6.33] | 6.15 [5.63,6.71] | 45.3 [42.2,48.6] | 92.4 [85.1,99.7] | 60.3 [55.3,65.2] | 4.43 [3.98,4.88] | 417.6 [393.7,441.6] | 154.6 [146.5,162.8]** |
| San Diego | 4.79 [4.48,5.12]** | 5.32 [4.83,5.86] | 40.8 [36.9,45.1] | 85.1 [80.5,89.7] | 57.3 [53.4,61.2] | 4.35 [4.02,4.68] | 404.9 [381.0,428.8]** | 161.1 [155.0,167.2] |

AM: Arithmetic mean; GM: Geometric mean

Overall p value for Hispanic/Latino background: LH (0.400), FSH (0.899), SHBG (0.021), E2 (0.386), Bio E2 (0.941), DHEAS (0.365), T (0.257), Bio T (0.780)

Overall p value for smoking status: LH (0.911), FSH (0.663), SHBG (0.010), E2 (0.777), Bio E2 (0.229), DHEAS (0.312), T (0.919), Bio T (0.022)

Overall p value for recruitment center: LH (0.001), FSH (0.672), SHBG (0.001), E2 (0.018), Bio E2 (0.331), DHEAS (0.763), T (0.012), Bio T (0.416)

Differences in mean hormone levels were evaluated for ordinal variables using overall tests. We conducted pairwise contrasts for nominal variables regardless of whether overall tests were significant or non-significant. When overall tests are not significant, pairwise contrasts should be interpreted with caution since post-hoc adjustment was not conducted.

** P<0.05

**Table S7. Associations^a^ of POPs serum concentrations with sex-related hormones, Hispanic/Latino postmenopausal women (N=716)**

| **POPs concentrations (ng/g Lipid)** | **LH (mIU/mL)** | **FSH (mIU/mL)** | **SHBG (nmol/L)**^c^ | **DHEAS (umol/L)** | **LH/FSH Ratio** | **Low E2** |
| --- | --- | --- | --- | --- | --- | --- |
|  | **β (95% CI)** | **β (95% CI)** | **β (95% CI)** | **β (95% CI)** | **β (95% CI)** | **OR (95% CI)** |
| **∑PBDEs** | -0.07 [-1.04,0.90] | -0.81 [-3.44,1.81] | 3.05 [-2.96,8.33] | 0.13 [-0.001,0.26]* | 0.01 [-0.01,0.03] | 0.96 [0.75,1.24] |
| **∑PCBs** | -0.33 [-1.69,1.02] | 0.52 [-2.17,3.21] | 7.25 [2.02,12.75]** | -0.12 [-0.29,0.05] | -0.02 [-0.05,0.01] | 0.85 [0.63,1.13] |
| **∑3MC-PCB inducers** | -0.33 [-1.67,1.02] | -0.56 [-3.12,2.01] | 3.05 [-1.98,9.42] | -0.08 [-0.26,0.10] | -0.01 [-0.04,0.01] | 0.90 [0.67,1.20] |
| **∑Dioxin-like PCBs** | -0.19 [-1.51,1.13] | -0.19 [-2.83,2.45] | 6.18 [0.10,11.6]** | -0.11 [-0.28,0.07] | -0.01 [-0.04,0.01] | 0.87 [0.65,1.16] |
| **∑Non-Dioxin-like PCBs** | -0.50 [-1.88,0.87] | 0.27 [-2.48,3.01] | 6.18 [1.01,11.6]** | -0.11 [-0.29,0.06] | -0.02 [-0.06,0.01] | 0.86 [0.64,1.16] |
| **∑PHB-PCB inducers** | -0.25 [-1.57,1.08] | 0.79 [-1.88,3.45] | 7.25 [2.02,12.8]** | -0.11 [-0.28,0.05] | -0.03 [-0.06,0.001] | 0.83 [0.62,1.11] |
| **PBB 153** | -0.69 [-1.99,0.62] | -2.14 [-4.94,0.65] | 3.05 [-3.92,10.5] | -0.02 [-0.17,0.14] | 0.004 [-0.02,0.03] | 1.00 [0.75,1.35] |
| **β-HCCH** | -1.03 [-2.57,0.50] | 0.67 [-1.85,3.19] | 11.6 [4.08,18.5]** | -0.16 [-0.38,0.06] | -0.03 [-0.05,-0.01]** | 0.90 [0.63,1.27] |
| **Mirex;^b^** non-detected vs <=median | -5.36 [-8.67,-2.04]** | -7.66 [-14.3,-1.05]** | 3.05 [-13.9,22.1] | -0.13 [-0.50,0.24] | -0.004 [-0.07,0.06] | 1.00 [0.40,2.48] |
| non-detected vs > median | -4.23 [-7.33,-1.13]** | -6.02 [-12.6,0.60] | -1.00 [-14.8,15.0] | -0.18 [-0.58,0.22] | -0.04 [-0.08,0.00] | 1.14 [0.52,2.49] |
| **HCB** | -0.81 [-2.38,0.77] | 0.02 [-3.28,3.32] | 8.33 [2.02,16.2]** | -0.06 [-0.29,0.16] | -0.01 [-0.04,0.01] | 0.78 [0.57,1.06] |
| **Oxychlordane** | -0.42 [-1.57,0.73] | 0.80 [-1.52,3.11] | 9.42 [4.08,15.0]** | -0.14 [-0.33,0.04] | -0.02 [-0.04,0.001]* | 1.08 [0.81,1.43] |
| **Trans-nonachlor** | -0.03 [-1.29,1.23] | 0.77 [-1.85,3.39] | 6.18 [1.01,11.6]** | -0.09 [-0.26,0.09] | -0.01 [-0.03,0.01] | 0.92 [0.69,1.22] |
| **p,p’-DDE** | -0.42 [-1.71,0.87] | -1.98 [-4.84,0.88] | 8.33 [2.02,13.9]** | 0.09 [-0.08,0.26] | 0.01 [-0.01,0.04] | 0.98 [0.71,1.36] |
| **p,p’-DDT;^b^** non-detected vs <=median | -1.99 [-4.76,0.77] | -0.69 [-5.98,4.60] | 3.05 [-9.52,16.2] | -0.02 [-0.36,0.32] | -0.002 [-0.07,0.06] | 2.64 [1.46,4.80]** |
| non-detected vs > median | -2.18 [-5.27,0.90] | -6.55 [-13.2,0.11] | 8.33 [-6.76,25.9] | 0.14 [-0.29,0.58] | 0.04 [-0.05,0.13] | 1.42 [0.70,2.87] |
| **o,p’-DDT;^b^** non-detected vs detected | 2.47 [-2.34,7.28] | -4.02 [-12.3,4.25] | 1.01 [-15.6,18.5] | -0.02 [-0.54,0.49] | 0.06 [-0.001,0.13] | 1.70 [0.58,4.95] |

^a^ Multivariable models adjusted for age, body mass index, waist-to-hip ratio, acculturation score Multiethnic Study of atherosclerosis, study sites, Hispanic/Latino background, educational attainment, estimated glomerular filtration rate, smoking status, alcohol consumption, physical activity levels, alternative healthy eating index 2010 and number of live births (women only)

^b^ POPs exposures were assessed from POPs serum concentration quartiles and interpreted as per quartile increase in concentration except for mirex, p,p’-DDT and o,p’-DDT. Mirex and p,p’-DDT were categorized as non-detected, <= median of detected values, > median of detected values while o,p’-DDT was categorized as detected vs non-detected. Median detected values were 2.7 ng/g lipid in men and 2.35 ng/g lipid in postmenopausal women for mirex, and 4.04 ng/g lipid in men and 8.59 ng/g lipid in postmenopausal women for p,p’-DDT. Only 7% of samples had o,p’-DDT values above LOD and this should be taken into consideration when interpreting findings

^c^ Ln transformed SHBG was modeled in postmenopausal women. Estimates for associations of POPs serum concentration with Ln transformed SHBG were back transformed $[100 \left( e^{\beta POPS}-1 \right)\%]$ and can be interpreted as a quartile increase in POPs concentration is associated with relative change in median or geometric mean of SHBG corresponding to $\beta\%$

Overall p value for Mirex: LH (0.004), FSH (0.043), SHBG (0.974), DHEAS (0.372), LH/FSH ratio (0.068), low E2 (0.776)

Overall p value for p,p’-DDT: LH (0.132), FSH (0.061), SHBG (0.284), DHEAS (0.559), LH/FSH ratio (0.416), low E2 (0.134)

∑PBDEs — PBDE 100, 153, 154, 17, 183, 209, 28, 47, 85, 99

∑PCBs — PCB 105, 114, 118, 138-158, 156, 153, 156, 157, 167, 170, 178, 180, 183, 187, 189, 194, 196-203, 199, 206, 209, 28, 66, 74, 99

∑3-methylcholanthrene (3MC) inducers: CYP IA inducers/substrates — PCB 66, 74, 105, 118, 156, 167

∑Dioxin-like PCBs — PCB 105, 114, 118, 156, 157, 167, 189

∑Non-Dioxin-like PCBs — PCB 28, 66, 74, 99, 138158, 146, 153, 170, 178, 180, 183, 187, 194, 196-203, 199, 206, 209

∑Phenobarbital-type (PB) inducers: CYP IIB inducers – PCB 99, 153, 180, 183, 187, 194, 196–203, 199, 206

** p<0.05; *p<0.10

**Table S8. Multivariable linear regression models evaluating associations^a^ of POPs serum concentrations in quartiles with sex-related hormones, Hispanic/Latino postmenopausal women (N=716)**

| **POPs concentrations (ng/g Lipid)^b^** | **LH (mIU/mL)** | | **FSH (mIU/mL)** | | **SHBG (nmol/L)^c^** | | **DHEAS (umol/L)** | | **LH/FSH Ratio** | |
| --- | --- | --- | --- | --- | --- | --- | --- | --- | --- | --- |
|  | **β (95% CI)** | **p for trend** | **β (95% CI)** | **p for trend** | **β (95% CI)** | **p for trend** | **β (95% CI)** | **p for trend** | **β (95% CI)** | **p for trend** |
| **∑PBDEs;** Q2 vs Q1 | -2.02 [-5.11,1.06] | 0.885 | 0.22 [-6.67,7.11] | 0.544 | -3.92 [-16.5,10.5] | 0.383 | 0.32 [-0.09,0.73] | **0.052** | -0.05 [-0.12,0.01] | 0.522 |
| Q3 vs Q1 | 0.27 [-3.24,3.78] |  | -1.01 [-8.20,6.18] |  | -3.92 [-18.1,13.9] |  | 0.26 [-0.15,0.66] |  | 0.01 [-0.07,0.08] |  |
| Q4 vs Q1 | -0.90 [-3.77,1.96] |  | -2.28 [-10.7,6.12] |  | 8.33 [-9.52,29.7] |  | 0.45 [0.04,0.86]** |  | 0.003 [-0.05,0.06] |  |
| **∑PCBs;** Q2 vs Q1 | 1.01 [-2.09,4.11] | 0.630 | 4.59 [-1.78,11.0] | 0.704 | 2.02 [-10.4,16.2] | **0.011** | -0.15 [-0.63,0.33] | 0.163 | -0.09 [-0.18,0.01] | 0.156 |
| Q3 vs Q1 | 1.28 [-2.23,4.80] |  | 3.91 [-4.14,12.0] |  | -1.00 [-15.6,15.0] |  | -0.11 [-0.62,0.40] |  | -0.07 [-0.17,0.03] |  |
| Q4 vs Q1 | -0.89 [-5.15,3.37] |  | 2.28 [-5.88,10.4] |  | 23.4 [5.13,43.3]** |  | -0.39 [-0.90,0.13] |  | -0.08 [-0.18,0.02] |  |
| **∑3MC inducers**; Q2 vs Q1 | -0.08 [-3.50,3.34] | 0.634 | 5.05 [-1.91,12.0] | 0.671 | -1.00 [-13.9,12.8] | 0.238 | -0.16 [-0.68,0.36] | 0.365 | -0.09 [-0.18,-0.01]** | 0.322 |
| Q3 vs Q1 | 1.55 [-2.04,5.15] |  | 1.71 [-5.11,8.53] |  | 0.20 [-14.8,17.4] |  | -0.25 [-0.76,0.25] |  | -0.05 [-0.13,0.04] |  |
| Q4 vs Q1 | -1.30 [-5.54,2.94] |  | 0.04 [-7.82,7.91] |  | 9.42 [-7.69,29.7] |  | -0.26 [-0.84,0.32] |  | -0.07 [-0.15,0.02] |  |
| **∑DL-PCBs;** Q2 vs Q1 | 2.08 [-1.21,5.36] | 0.776 | 5.49 [-1.65,12.6] | 0.887 | 2.02 [-10.4,17.4] | **0.034** | -0.06 [-0.57,0.45] | 0.249 | -0.07 [-0.16,0.02] | 0.263 |
| Q3 vs Q1 | 1.41 [-2.53,5.36] |  | 2.76 [-4.81,10.3] |  | 3.05 [-11.3,19.7] |  | -0.28 [-0.79,0.23] |  | -0.06 [-0.14,0.03] |  |
| Q4 vs Q1 | 0.07 [-4.00,4.14] |  | 1.16 [-6.68,8.99] |  | 18.5 [1.01,39.1]** |  | -0.29 [-0.86,0.28] |  | -0.06 [-0.14,0.03] |  |
| **∑NDL-PCBs;** Q2 vs Q1 | 0.63 [-2.51,3.78] | 0.471 | 3.76 [-2.65,10.2] | 0.848 | 3.05 [-9.52,17.4] | **0.016** | -0.18 [-0.65,0.29] | 0.209 | -0.09 [-0.18,0.01] | 0.200 |
| Q3 vs Q1 | 1.56 [-1.88,5.00] |  | 4.23 [-3.49,11.9] |  | -0.10 [-14.8,17.4] |  | -0.18 [-0.69,0.32] |  | -0.07 [-0.16,0.03] |  |
| Q4 vs Q1 | -1.76 [-6.05,2.52] |  | 0.98 [-7.44,9.40] |  | 22.1 [5.13,43.3]** |  | -0.37 [-0.91,0.18] |  | -0.08 [-0.19,0.03] |  |
| **∑PHB-PCB inducers;** Q2 vs Q1 | 0.60 [-2.63,3.84] | 0.713 | 3.05 [-3.54,9.64] | 0.563 | 7.25 [-4.88,22.1] | **0.008** | -0.05 [-0.54,0.44] | 0.176 | -0.08 [-0.18,0.02] | 0.091 |
| Q3 vs Q1 | 0.72 [-2.84,4.29] |  | 4.29 [-3.68,12.3] |  | -1.98 [-16.5,13.9] |  | -0.19 [-0.69,0.31] |  | -0.07 [-0.18,0.03] |  |
| Q4 vs Q1 | -0.74 [-4.88,3.40] |  | 2.47 [-5.77,10.7] |  | 27.1 [9.40,49.2]** |  | -0.33 [-0.85,0.19] |  | -0.10 [-0.20,0.01] |  |
| **PBB 153;** Q2 vs Q1 | 2.47 [-1.54,6.48] | 0.300 | 4.72 [-5.33,14.77] | 0.133 | 6.18 [-10.4,25.9] | 0.406 | 0.24 [-0.29,0.76] | 0.825 | 0.02 [-0.04,0.08] | 0.749 |
| Q3 vs Q1 | 0.75 [-3.30,4.80] |  | -1.15 [-8.79,6.48] |  | -1.00 [-18.1,19.7] |  | -0.03 [-0.51,0.46] |  | 0.02 [-0.06,0.09] |  |
| Q4 vs Q1 | -1.33 [-5.43,2.77] |  | -4.48 [-13.4,4.41] |  | 12.8 [-9.52,39.1] |  | 0.05 [-0.45,0.54] |  | 0.02 [-0.05,0.08] |  |
| **β-HCCH;** Q2 vs Q1 | 0.20 [-3.45,3.84] | 0.185 | 1.87 [-5.78,9.53] | 0.603 | 4.08 [-9.52,18.5] | **0.001** | -0.11 [-0.57,0.36] | 0.163 | -0.06 [-0.14,0.02] | **0.008** |
| Q3 vs Q1 | -1.87 [-5.58,1.85] |  | 4.42 [-3.27,12.1] |  | 18.5 [0.10,39.1]** |  | -0.31 [-0.87,0.26] |  | -0.11 [-0.19,-0.04]** |  |
| Q4 vs Q1 | -2.68 [-7.33,1.97] |  | 1.61 [-6.66,9.88] |  | 35.0 [12.8,61.6]** |  | -0.46 [-1.15,0.23] |  | -0.09 [-0.16,-0.02]** |  |
| **HCB;** Q2 vs Q1 | 0.04 [-3.56,3.63] | 0.315 | 2.87 [-3.91,9.66] | 0.989 | 7.25 [-6.76,23.4] | **0.014** | -0.11 [-0.53,0.32] | 0.591 | -0.08 [-0.15,-0.001]** | 0.331 |
| Q3 vs Q1 | -2.61 [-6.65,1.43] |  | -0.98 [-8.47,6.51] |  | 19.7 [0.10,41.9]** |  | -0.15 [-0.67,0.37] |  | -0.04 [-0.12,0.03] |  |
| Q4 vs Q1 | -1.32 [-6.08,3.45] |  | 1.88 [-9.73,13.5] |  | 27.1 [3.05,55.3]** |  | -0.18 [-0.87,0.51] |  | -0.05 [-0.12,0.03] |  |
| **Oxychlordane;** Q2 vs Q1 | -1.10 [-4.52,2.32] | 0.476 | -2.91 [-9.88,4.07] | 0.499 | 1.01 [-10.4,13.9] | **0.0001** | -0.14 [-0.60,0.33] | 0.132 | -0.01 [-0.08,0.07] | **0.076** |
| Q3 vs Q1 | -0.41 [-3.71,2.88] |  | 0.70 [-6.23,7.62] |  | 11.6 [-4.88,31.0] |  | -0.31 [-0.76,0.14] |  | -0.04 [-0.10,0.03] |  |
| Q4 vs Q1 | -1.47 [-5.16,2.21] |  | 1.63 [-5.55,8.80] |  | 31.0 [12.8,52.2]** |  | -0.43 [-1.01,0.16] |  | -0.05 [-0.12,0.02] |  |
| **Trans-nonachlor;** Q2 vs Q1 | -0.11 [-3.44,3.22] | 0.964 | -2.30 [-9.25,4.65] | 0.564 | 8.33 [-4.88,22.1] | **0.022** | 0.26 [-0.17,0.69] | 0.334 | 0.001 [-0.07,0.08] | 0.350 |
| Q3 vs Q1 | 0.0001 [-3.52,3.52] |  | -0.04 [-7.23,7.15] |  | 1.01 [-13.1,17.4] |  | -0.20 [-0.63,0.23] |  | -0.02 [-0.08,0.05] |  |
| Q4 vs Q1 | -0.13 [-4.10,3.84] |  | 1.44 [-6.62,9.49] |  | 23.4 [5.13,44.8]** |  | -0.11 [-0.63,0.40] |  | -0.02 [-0.09,0.05] |  |
| **p,p’-DDE;** Q2 vs Q1 | 1.93 [-1.13,4.98] | 0.522 | 1.95 [-5.30,9.19] | 0.174 | -3.92 [-16.5,11.6] | **0.005** | -0.17 [-0.58,0.24] | 0.297 | 0.01 [-0.05,0.07] | 0.409 |
| Q3 vs Q1 | 0.89 [-2.68,4.46] |  | -1.31 [-8.36,5.74] |  | 4.08 [-7.69,16.2] |  | 0.08 [-0.40,0.57] |  | 0.01 [-0.06,0.08] |  |
| Q4 vs Q1 | -0.94 [-4.91,3.02] |  | -5.30 [-14.9,4.26] |  | 25.9 [7.25,46.2]** |  | 0.19 [-0.36,0.74] |  | 0.04 [-0.04,0.12] |  |

^a^ Multivariable models adjusted for age, body mass index, waist-to-hip ratio, acculturation score Multiethnic Study of atherosclerosis, study sites, Hispanic/Latino background, educational attainment, estimated glomerular filtration rate, smoking status, alcohol consumption, physical activity levels, alternative healthy eating index 2010 and number of live births (women only). We did not evaluate low E2 in women due to small subgroup size.

^b^ POPs concentrations were evaluated in quartiles as follows - Q4: quartile 4; Q3: quartile 3; Q2: quartile 2; Q1: quartile 1

^c^ Ln transformed SHBG was modeled in postmenopausal women. Estimates for associations of POPs serum concentration with Ln transformed SHBG were back transformed $[100 \left( e^{\beta POPS}-1 \right)\%]$ and can be interpreted as a quartile increase in POPs concentration is associated with relative change in median or geometric mean of SHBG corresponding to $\beta\%$

∑PBDEs — PBDE 100, 153, 154, 17, 183, 209, 28, 47, 85, 99

∑PCBs — PCB 105, 114, 118, 138-158, 156, 153, 156, 157, 167, 170, 178, 180, 183, 187, 189, 194, 196-203, 199, 206, 209, 28, 66, 74, 99

∑3-methylcholanthrene (3MC) inducers: CYP IA inducers/substrates — PCB 66, 74, 105, 118, 156, 167

∑Dioxin-like PCBs — PCB 105, 114, 118, 156, 157, 167, 189

∑Non-Dioxin-like PCBs — PCB 28, 66, 74, 99, 138158, 146, 153, 170, 178, 180, 183, 187, 194, 196-203, 199, 206, 209

∑Phenobarbital-type (PB) inducers: CYP IIB inducers – PCB 99, 153, 180, 183, 187, 194, 196–203, 199, 206

** p<0.05; *p<0.10

**Table S9. Multivariable linear regression models evaluating associations^a^ of select PBDE congeners with DHEAS in postmenopausal women (N=716)**

| **POPs concentrations (ng/g Lipid)** | **DHEAS (umol/L)** | |
| --- | --- | --- |
|  | **β (95% CI)** | **p for trend** |
| **PBDE 100**; [quartile increase] | 0.15 [0.01,0.28]** | **0.033** |
| Q3 vs Q1 | 0.20 [-0.18,0.59] |  |
| Q3 vs Q1 | 0.26 [-0.16,0.69] |  |
| Q4 vs Q1 | 0.47 [0.04,0.90]** |  |
| **PBDE 153**; [quartile increase] | 0.08 [-0.07,0.23] | 0.292 |
| Q3 vs Q1 | -0.003 [-0.42,0.42] |  |
| Q3 vs Q1 | 0.39 [-0.14,0.92] |  |
| Q4 vs Q1 | 0.16 [-0.31,0.64] |  |
| **PBDE 99**; [quartile increase] | 0.09 [-0.05,0.23] | 0.198 |
| Q3 vs Q1 | 0.18 [-0.23,0.59] |  |
| Q3 vs Q1 | 0.07 [-0.34,0.48] |  |
| Q4 vs Q1 | 0.32 [-0.11,0.75] |  |
| **PBDE 47**; [quartile increase] | 0.14 [0.02,0.27]** | **0.022** |
| Q3 vs Q1 | 0.43 [0.04,0.81]** |  |
| Q3 vs Q1 | 0.40 [-0.01,0.80]* |  |
| Q4 vs Q1 | 0.48 [0.09,0.87]** |  |

^a^ Multivariable models adjusted for age, body mass index, waist-to-hip ratio, acculturation score Multiethnic Study of atherosclerosis, study sites, Hispanic/Latino background, educational attainment, estimated glomerular filtration rate, smoking status, alcohol consumption, physical activity levels, alternative healthy eating index 2010 and number of live births (women only)

** p<0.05; *p<0.10

**Table S10. Multivariable linear regression models evaluating associations^a^ of POPs serum concentrations with sex-related hormones, Hispanic/Latino men (N=1,073)**

| **POPs concentrations (ng/g Lipid)^b^** | **LH (mIU/mL)^c^** | **FSH (mIU/mL)^c^** | **E2 (pmol/L)** | **Bio E2 (pmol/L)** | **SHBG (nmol/L)^c^** | **DHEAS (umol/L)** |
| --- | --- | --- | --- | --- | --- | --- |
|  | **β (95% CI)** | **β (95% CI)** | **β (95% CI)** | **β (95% CI)** | **β (95% CI)** | **β (95% CI)** |
| **∑PBDEs** | -0.30 [-3.92,4.08] | 1.01 [-3.92,5.13] | 0.71 [-2.92,4.34] | 0.11 [-2.14,2.35] | 2.02 [-1.00,6.18] | 0.01 [-0.13,0.15] |
| **∑PCBs** | -1.98 [-6.76,3.05] | 3.05 [-2.96,9.42] | -6.36 [-10.7,-2.02]** | -4.48 [-7.22,-1.73]** | 0.40 [-2.96,4.08] | 0.06 [-0.11,0.23] |
| **∑3MC-PCB inducers** | -1.98 [-5.82,2.02] | 2.02 [-3.92,7.25] | -4.68 [-8.78,-0.57]** | -3.36 [-6.02,-0.71]** | 1.01 [-1.98,5.13] | -0.04 [-0.23,0.15] |
| **∑Dioxin-like PCBs** | -1.98 [-5.82,3.05] | 2.02 [-2.96,8.33] | -4.71 [-8.82,-0.60]** | -3.46 [-6.13,-0.79]** | 2.02 [-1.98,5.13] | -0.05 [-0.23,0.14] |
| **∑Non-Dioxin-like PCBs** | -2.96 [-6.76,2.02] | 2.02 [-3.92,7.25] | -6.76 [-11.2,-2.29]** | -4.66 [-7.43,-1.90]** | 0.30 [-2.96,4.08] | 0.12 [-0.07,0.31] |
| **∑PHB-PCB inducers** | -2.96 [-7.69,1.01] | 1.01 [-3.92,7.25] | -8.73 [-13.5,-3.94]** | -5.67 [-8.48,-2.87]** | 0.10 [-3.92,4.08] | 0.02 [-0.15,0.19] |
| **PBB 153** | -1.98 [-5.82,3.05] | -3.92 [-9.52,2.02] | 0.73 [-3.88,5.34] | -0.25 [-3.38,2.89] | 1.01 [-3.92,5.13] | 0.07 [-0.17,0.31] |
| **β-HCCH** | -0.30 [-5.82,5.13] | 1.01 [-5.82,7.25] | -4.80 [-8.80,-0.80]** | -2.68 [-5.43,0.07] | -2.96 [-5.82,1.01] | -0.20 [-0.42,0.03] |
| **Mirex;** non-detected vs <=median | -1.98 [-12.2,8.33] | 0.20 [-13.1,15.0] | -10.1 [-19.2,-0.97]** | -4.18 [-9.75,1.39] | -10.4 [-18.1,-1.98]** | 0.44 [-0.07,0.95] |
| non-detected vs > median | -2.96 [-13.9,9.42] | 5.13 [-9.52,23.4] | -16.3 [-27.7,-4.94]** | -10.1 [-16.9,-3.44]** | -3.92 [-13.1,6.18] | 0.20 [-0.26,0.65] |
| **HCB** | -1.98 [-8.61,4.08] | 0.30 [-5.82,7.25] | -3.58 [-8.55,1.39] | -1.62 [-4.81,1.56] | -1.98 [-4.88,2.02] | 0.08 [-0.16,0.31] |
| **Oxychlordane** | -1.00 [-4.88,4.08] | 1.01 [-4.88,7.25] | -5.08 [-8.11,-2.05]** | -4.23 [-6.17,-2.28]** | 3.05 [-1.00,6.18] | -0.02 [-0.23,0.19] |
| **Trans-nonachlor** | -1.00 [-5.82,3.05] | 3.05 [-2.96,8.33] | -3.06 [-6.51,0.39]* | -2.75 [-4.91,-0.58]** | 2.02 [-1.98,5.13] | 0.002 [-0.18,0.19] |
| **p,p’-DDE** | -3.92 [-8.61,1.01] | 2.02 [-4.88,8.33] | -3.85 [-7.70,0.01]* | -2.82 [-5.23,-0.41]** | 2.02 [-1.98,6.18] | -0.11 [-0.32,0.10] |
| **p,p’-DDT;** non-detected vs <=median | 4.08 [-7.69,17.4] | 16.2 [-1.98,37.7] | -18.8 [-28.7,-8.94]** | -12.2 [-18.0,-6.37]** | -6.76 [-14.8,2.02] | 0.26 [-0.30,0.81] |
| non-detected vs > median | 1.01 [-10.4,12.8] | 13.9 [-1.00,29.7] | -14.1 [-22.4,-5.77]** | -11.0 [-16.7,-5.43]** | 8.33 [-1.00,17.4] | -0.22 [-0.66,0.22] |
| **o,p’-DDT;** non-detected vs detected | -16.5 [-34.3,7.25] | -12.2 [-33.6,15.0] | -14.1 [-29.1,0.93] | -9.46 [-19.3,0.32] | 5.13 [-9.52,22.1] | -0.47 [-1.08,0.14] |
|  | **T (ng/dL)** | **Bio T (ng/dL)** | **LH/FSH Ratio** | **E2/T Ratio** | **Bio E2/Bio T Ratio** |  |
|  | **β (95% CI)** | **β (95% CI)** | **β (95% CI)** | **β (95% CI)** | **β (95% CI)** |  |
| **∑PBDEs** | 2.23 [-8.77,13.2] | 0.66 [-2.83,4.16] | -0.01 [-0.05,0.04] | 0.01 [-0.01,0.02] | 0.02 [-0.02,0.05] |  |
| **∑PCBs** | -7.04 [-22.3,8.19] | -3.28 [-7.18,0.62] | -0.05 [-0.10,0.001]* | -0.01 [-0.03,0.02] | -0.002 [-0.05,0.05] |  |
| **∑3MC-PCB inducers** | -1.10 [-16.1,13.9] | -0.74 [-4.87,3.39] | -0.04 [-0.09,0.01] | -0.001 [-0.03,0.03] | 0.01 [-0.05,0.07] |  |
| **∑Dioxin-like PCBs** | 0.46 [-14.3,15.2] | -0.61 [-4.65,3.42] | -0.04 [-0.08,0.01] | -0.003 [-0.03,0.02] | 0.01 [-0.05,0.06] |  |
| **∑Non-Dioxin-like PCBs** | -5.12 [-20.1,9.81] | -2.36 [-6.20,1.47] | -0.04 [-0.09,0.01] | -0.01 [-0.03,0.01] | -0.01 [-0.06,0.04] |  |
| **∑PHB-PCB inducers** | -10.1 [-25.6,5.37] | -3.56 [-7.36,0.24] | -0.04 [-0.09,0.02] | -0.01 [-0.03,0.01] | -0.02 [-0.06,0.03] |  |
| **PBB 153** | 8.04 [-4.91,21.0] | 1.53 [-2.36,5.41] | 0.04 [-0.02,0.09] | -0.0003 [-0.01,0.01] | 0.002 [-0.02,0.03] |  |
| **β-HCCH** | -29.4 [-45.6,-13.2]** | -7.78 [-12.8,-2.74]** | -0.02 [-0.07,0.03] | 0.02 [-0.02,0.06] | 0.04 [-0.04,0.13] |  |
| **Mirex;** non-detected vs <=median | -19.9 [-50.0,10.2] | 1.43 [-7.46,10.3] | -0.04 [-0.15,0.08] | -0.01 [-0.04,0.01] | -0.04 [-0.09,0.01] |  |
| non-detected vs > median | -24.1 [-57.8,9.49] | -5.56 [-16.7,5.58] | -0.04 [-0.18,0.10] | -0.002 [-0.05,0.05] | -0.0003 [-0.10,0.10] |  |
| **HCB** | -3.75 [-19.5,12.0] | 1.98 [-3.75,7.70] | -0.03 [-0.09,0.03] | -0.01 [-0.03,0.01] | -0.02 [-0.05,0.02] |  |
| **Oxychlordane** | -6.01 [-20.3,8.24] | -3.93 [-8.19,0.34]* | -0.01 [-0.06,0.05] | -0.01 [-0.03,0.01] | -0.004 [-0.05,0.05] |  |
| **Trans-nonachlor** | -1.81 [-16.1,12.5] | -1.99 [-6.04,2.07] | -0.04 [-0.09,0.01] | -0.01 [-0.03,0.02] | -0.001 [-0.05,0.05] |  |
| **p,p’-DDE** | -11.4 [-26.2,3.40] | -3.5 [-8.06,1.05] | -0.06 [-0.11,-0.01]** | 0.01 [-0.02,0.04] | 0.03 [-0.04,0.10] |  |
| **p,p’-DDT;** non-detected vs <=median | -56.8 [-92.5,-21.1]** | -19.3 [-28.5,-10.2]** | -0.09 [-0.22,0.04] | -0.02 [-0.04,0.01] | -0.03 [-0.08,0.01] |  |
| non-detected vs > median | -29.0 [-60.0,1.93] | -16.5 [-27.2,-5.82]** | -0.12 [-0.23,-0.02]** | 0.02 [-0.06,0.09] | 0.06 [-0.11,0.22] |  |
| **o,p’-DDT;** non-detected vs detected | -71.2 [-126.4,-16.1]** | -25.9 [-47.1,-4.66]** | -0.01 [-0.23,0.20] | 0.01 [-0.03,0.05] | 0.03 [-0.05,0.11] |  |

^a^ Multivariable models adjusted for age, body mass index, waist-to-hip ratio, acculturation score Multiethnic Study of atherosclerosis, study sites, Hispanic/Latino background, educational attainment, estimated glomerular filtration rate, smoking status, alcohol consumption, physical activity levels, and alternative healthy eating index 2010

^b^ POPs exposures were assessed from POPs serum concentration quartiles and interpreted as per quartile increase in concentration except for mirex, p,p’-DDT and o,p’-DDT. Mirex and p,p’-DDT were categorized as non-detected, <= median of detected values, > median of detected values while o,p’-DDT was categorized as detected vs non-detected. Median detected values were 2.7 ng/g lipid in men and 2.35 ng/g lipid in postmenopausal women for mirex, and 4.04 ng/g lipid in men and 8.59 ng/g lipid in postmenopausal women for p,p’-DDT. Only 7% of samples had o,p’-DDT values above LOD and this should be taken into consideration when interpreting findings

^c^ Ln transformed SHBG, LH and FSH were modeled in men. Estimates for associations of POPs serum concentration with Ln transformed hormones were back transformed $[100 \left( e^{\beta POPS}-1 \right)\%]$ and can be interpreted as a quartile increase in POPs concentration is associated with relative change in median or geometric mean of hormone corresponding to $\beta\%$

Overall p value for Mirex: LH (0.657), FSH (0.498), E2 (0.005), Bio E2 (0.003), SHBG (0.382), DHEAS (0.345), T (0.147), Bio T (0.347), LH/FSH ratio (0.546), E2/T ratio (0.916), Bio E2/Bio T ratio (0.960)

Overall p value for p,p’-DDT: LH (0.775), FSH (0.031), E2 (0.0001), Bio E2 (<0.0001), SHBG (0.267), DHEAS (0.557), T (0.009), Bio T (0.002), LH/FSH ratio (0.015), E2/T ratio (0.781), Bio E2/Bio T ratio (0.589)

∑PBDEs — PBDE 100, 153, 154, 17, 183, 209, 28, 47, 85, 99

∑PCBs — PCB 105, 114, 118, 138-158, 156, 153, 156, 157, 167, 170, 178, 180, 183, 187, 189, 194, 196-203, 199, 206, 209, 28, 66, 74, 99

∑3-methylcholanthrene (3MC) inducers: CYP IA inducers/substrates — PCB 66, 74, 105, 118, 156, 167

∑Dioxin-like PCBs — PCB 105, 114, 118, 156, 157, 167, 189

∑Non-Dioxin-like PCBs — PCB 28, 66, 74, 99, 138158, 146, 153, 170, 178, 180, 183, 187, 194, 196-203, 199, 206, 209

∑Phenobarbital-type (PB) inducers: CYP IIB inducers – PCB 99, 153, 180, 183, 187, 194, 196–203, 199, 206

** p<0.05; *p<0.10

**Table S11. Multivariable linear regression models evaluating associations^a^ of POPs serum concentrations in quartiles with sex-related hormones, Hispanic/Latino men (N=1,073)**

| **POPs concentrations (ng/g Lipid)^b^** | **LH (mIU/mL)^c^** | | **FSH (mIU/mL)^c^** | | **E2 (pmol/L)** | | **Bio E2 (pmol/L)** | | **SHBG (nmol/L)^c^** | | **DHEAS (umol/L)** | |
| --- | --- | --- | --- | --- | --- | --- | --- | --- | --- | --- | --- | --- |
|  | **β (95% CI)** | **p for trend** | **β (95% CI)** | **p for trend** | **β (95% CI)** | **p for trend** | **β (95% CI)** | **p for trend** | **β (95% CI)** | **p for trend** | **β (95% CI)** | **p for trend** |
| **∑PBDEs;** Q2 vs Q1 | -8.61 [-18.9,3.05] | 0.889 | -1.98 [-18.1,18.5] | 0.843 | -1.50 [-11.7,8.72] | 0.702 | 0.54 [-6.10,7.17] | 0.926 | 1.01 [-8.61,11.6] | 0.194 | 0.06 [-0.46,0.58] | 0.888 |
| Q3 vs Q1 | -5.82 [-18.1,7.25] |  | 6.18 [-9.52,25.9] |  | -7.66 [-16.8,1.48] |  | -5.22 [-11.3,0.91] |  | 5.13 [-4.88,16.2] |  | 0.02 [-0.51,0.54] |  |
| Q4 vs Q1 | -1.00 [-12.2,10.5] |  | -1.98 [-15.6,15.0] |  | 5.15 [-6.72,17.0] |  | 2.73 [-4.21,9.67] |  | 6.18 [-3.92,17.4] |  | 0.05 [-0.38,0.48] |  |
| **∑PCBs;** Q2 vs Q1 | -2.96 [-14.8,10.5] | 0.377 | 8.33 [-4.88,24.6] | 0.271 | -4.79 [-16.2,6.64] | **0.004** | -1.97 [-10.1,6.11] | **0.001** | -5.82 [-15.6,4.08] | 0.817 | 0.41 [-0.12,0.94] | 0.494 |
| Q3 vs Q1 | 1.01 [-11.3,15.0] |  | 17.4 [0.10,36.3]** |  | -9.41 [-23.2,4.35] |  | -8.34 [-17.3,0.60] |  | 4.08 [-6.76,16.2] |  | 0.44 [-0.10,0.97] |  |
| Q4 vs Q1 | -7.69 [-19.8,7.25] |  | 10.5 [-5.82,31.0] |  | -19.0 [-33.0,-4.95]** |  | -12.5 [-21.4,-3.62]** |  | -1.98 [-12.2,9.42] |  | 0.26 [-0.28,0.81] |  |
| **∑3MC inducers**; Q2 vs Q1 | -1.00 [-12.2,10.5] | 0.376 | -1.98 [-14.8,13.9] | 0.585 | -4.59 [-15.6,6.40] | **0.026** | -3.30 [-10.7,4.14] | **0.013** | 2.02 [-8.61,12.8] | 0.439 | -0.46 [-1.02,0.09] | 0.668 |
| Q3 vs Q1 | 4.08 [-8.61,17.4] |  | 17.4 [-0.10,37.7]* |  | -8.30 [-20.7,4.11] |  | -7.33 [-15.8,1.16] |  | 5.13 [-6.76,17.4] |  | 0.16 [-0.50,0.81] |  |
| Q4 vs Q1 | -6.76 [-18.1,6.18] |  | 1.01 [-15.6,19.7] |  | -14.3 [-27.3,-1.18]** |  | -9.93 [-18.4,-1.50]** |  | 4.08 [-6.76,16.2] |  | -0.32 [-0.92,0.28] |  |
| **∑DL-PCBs;** Q2 vs Q1 | -6.76 [-17.3,6.18] | 0.498 | -4.88 [-18.1,9.42] | 0.422 | -6.49 [-17.4,4.38] | **0.025** | -4.42 [-11.9,3.02] | **0.011** | 2.02 [-8.61,12.8] | 0.386 | -0.25 [-0.80,0.31] | 0.629 |
| Q3 vs Q1 | 4.08 [-8.61,17.4] |  | 17.4 [-0.10,37.7]* |  | -9.79 [-22.4,2.79] |  | -8.53 [-17.1,0.06] |  | 5.13 [-6.76,17.4] |  | 0.13 [-0.50,0.76] |  |
| Q4 vs Q1 | -7.69 [-18.9,6.18] |  | 1.01 [-13.9,19.7] |  | -14.7 [-27.7,-1.68]** |  | -10.4 [-18.8,-1.86]** |  | 4.08 [-6.76,16.2] |  | -0.26 [-0.85,0.33] |  |
| **∑NDL-PCBs;** Q2 vs Q1 | -1.98 [-13.1,11.6] | 0.219 | 8.33 [-5.82,24.6] | 0.561 | -4.91 [-16.3,6.48] | **0.003** | -2.13 [-10.2,5.91] | **0.001** | -5.82 [-15.6,5.13] | 0.852 | 0.36 [-0.17,0.89] | 0.216 |
| Q3 vs Q1 | 3.05 [-10.4,17.4] |  | 18.5 [0.10,40.5]** |  | -9.10 [-22.0,3.78] |  | -8.64 [-17.2,-0.03]** |  | 6.18 [-4.88,18.5] |  | 0.14 [-0.36,0.64] |  |
| Q4 vs Q1 | -8.61 [-21.3,5.13] |  | 5.13 [-9.52,22.1] |  | -20.3 [-34.9,-5.72]** |  | -13.1 [-22.1,-4.05]** |  | -1.98 [-13.1,9.42] |  | 0.48 [-0.12,1.07] |  |
| **∑PHB-PCB inducers;** Q2 vs Q1 | -2.96 [-14.8,10.5] | 0.187 | 4.08 [-10.42,20.9] | 0.690 | -3.35 [-14.7,8.00] | **0.0001** | -1.37 [-9.50,6.75] | **0.0001** | -6.76 [-16.5,4.08] | 0.958 | 0.04 [-0.57,0.65] | 0.793 |
| Q3 vs Q1 | 1.01 [-12.2,15.0] |  | 9.42 [-7.69,31.0] |  | -14.4 [-27.8,-0.99]** |  | -10.6 [-19.4,-1.86]** |  | 2.02 [-8.61,12.8] |  | 0.06 [-0.50,0.61] |  |
| Q4 vs Q1 | -10.4 [-22.1,4.08] |  | 3.05 [-12.2,20.9] |  | -24.5 [-39.6,-9.30]** |  | -15.2 [-24.6,-5.89]** |  | -2.96 [-13.9,8.33] |  | 0.07 [-0.51,0.65] |  |
| **PBB 153;** Q2 vs Q1 | -7.69 [-18.9,4.08] | 0.485 | -17.3 [-30.9,-1.98]** | 0.160 | 14.7 [2.55,26.8]** | 0.756 | 6.89 [-1.10,14.9] | 0.877 | 10.5 [0.10,22.1]** | 0.714 | 0.45 [-0.18,1.07] | 0.560 |
| Q3 vs Q1 | -8.61 [-20.6,6.18] |  | -12.2 [-29.5,9.42] |  | 15.0 [-0.83,30.9] |  | 4.69 [-4.38,13.8] |  | 13.9 [1.01,28.4]** |  | 0.29 [-0.48,1.07] |  |
| Q4 vs Q1 | -6.76 [-18.1,7.25] |  | -17.3 [-31.6,1.01] |  | 5.71 [-8.36,19.8] |  | 1.44 [-8.42,11.3] |  | 5.13 [-8.61,19.7] |  | 0.35 [-0.34,1.05] |  |
| **β-HCCH;** Q2 vs Q1 | 1.01 [-12.2,15.0] | 0.926 | -0.30 [-17.3,19.7] | 0.867 | -11.9 [-22.5,-1.27]** | **0.019** | -5.74 [-12.1,0.58] | 0.056 | -9.52 [-17.3,-1.00]** | 0.222 | 0.12 [-0.42,0.67] | 0.083 |
| Q3 vs Q1 | -1.98 [-16.5,16.2] |  | 0.20 [-18.1,22.1] |  | -16.5 [-27.5,-5.54]** |  | -8.18 [-15.3,-1.05]** |  | -11.3 [-20.6,-1.00]** |  | -0.33 [-0.96,0.31] |  |
| Q4 vs Q1 | -0.30 [-15.6,18.5] |  | 2.02 [-17.3,25.9] |  | -15.0 [-27.6,-2.50]** |  | -8.37 [-16.7,0.01] |  | -7.69 [-18.1,4.08] |  | -0.50 [-1.20,0.20] |  |
| **HCB;** Q2 vs Q1 | -1.98 [-13.9,12.8] | 0.473 | 4.08 [-13.9,24.6] | 0.939 | -3.60 [-14.9,7.72] | 0.158 | 0.23 [-7.48,7.94] | 0.317 | -11.3 [-20.6,-0.10]** | 0.359 | -0.11 [-0.59,0.37] | 0.534 |
| Q3 vs Q1 | 2.02 [-12.2,19.7] |  | 3.05 [-15.6,25.9] |  | -8.43 [-19.4,2.58] |  | -3.37 [-9.91,3.16] |  | -5.82 [-13.1,3.05] |  | 0.18 [-0.49,0.84] |  |
| Q4 vs Q1 | -13.1 [-28.8,6.18] |  | -1.00 [-17.3,19.7] |  | -9.44 [-26.6,7.72] |  | -4.23 [-15.9,7.39] |  | -4.88 [-15.6,6.18] |  | 0.15 [-0.49,0.78] |  |
| **Oxychlordane;** Q2 vs Q1 | -3.92 [-14.8,7.25] | 0.823 | 2.02 [-13.1,18.5] | 0.661 | -8.28 [-16.0,-0.57]** | **0.001** | -6.62 [-12.3,-0.91]** | **0.0001** | 5.13 [-5.82,16.2] | 0.100 | -0.15 [-0.70,0.40] | 0.828 |
| Q3 vs Q1 | -3.92 [-14.8,9.42] |  | 3.05 [-12.2,20.9] |  | -12.4 [-22.7,-2.18]** |  | -10.2 [-17.0,-3.31]** |  | 10.5 [-1.00,23.4] |  | -0.34 [-0.90,0.22] |  |
| Q4 vs Q1 | -1.98 [-14.8,12.8] |  | 4.08 [-14.8,27.1] |  | -15.7 [-25.5,-5.81]** |  | -13.0 [-19.3,-6.74]** |  | 8.33 [-2.96,20.9] |  | -0.01 [-0.68,0.66] |  |
| **Trans-nonachlor;** Q2 vs Q1 | -3.92 [-15.6,9.42] | 0.521 | 10.5 [-3.92,28.4] | 0.368 | -3.00 [-12.0,5.96] | **0.082** | -2.94 [-8.86,2.97] | **0.013** | 5.13 [-4.88,15.0] | 0.351 | -0.13 [-0.69,0.44] | 0.982 |
| Q3 vs Q1 | -5.82 [-17.3,6.18] |  | 8.33 [-5.82,23.4] |  | -6.80 [-17.5,3.91] |  | -6.00 [-13.1,1.13] |  | 7.25 [-4.88,19.7] |  | -0.07 [-0.65,0.52] |  |
| Q4 vs Q1 | -3.92 [-17.3,11.6] |  | 10.5 [-7.69,31.0] |  | -8.92 [-19.6,1.72] |  | -8.15 [-14.6,-1.72]** |  | 5.13 [-5.82,16.2] |  | -0.02 [-0.62,0.58] |  |
| **p,p’-DDE;** Q2 vs Q1 | -4.88 [-15.6,7.25] | 0.141 | -1.00 [-15.6,17.4] | 0.678 | -9.53 [-20.1,1.00] | **0.051** | -4.21 [-11.1,2.70] | **0.022** | -1.98 [-11.3,7.25] | 0.248 | 0.72 [-0.05,1.49] | 0.309 |
| Q3 vs Q1 | -6.76 [-17.3,6.18] |  | 1.01 [-14.8,19.7] |  | -13.6 [-24.9,-2.23]** |  | -7.53 [-14.7,-0.35]** |  | -1.00 [-11.3,9.42] |  | -0.01 [-0.65,0.63] |  |
| Q4 vs Q1 | -11.3 [-24.4,4.08] |  | 4.08 [-16.5,29.7] |  | -12.6 [-24.7,-0.38]** |  | -8.61 [-16.0,-1.22]** |  | 6.18 [-4.88,18.5] |  | -0.04 [-0.76,0.68] |  |
|  | **T (ng/dL)** | | **Bio T (ng/dL)** | | **LH/FSH Ratio** | | **E2/T Ratio** | | **Bio E2/Bio T Ratio** | |  |  |
|  | **β (95% CI)** | **p for trend** | **β (95% CI)** | **p for trend** | **β (95% CI)** | **p for trend** | **β (95% CI)** | **p for trend** | **β (95% CI)** | **p for trend** |  |  |
| **∑PBDEs;** Q2 vs Q1 | -3.95 [-42.5,34.6] | 0.690 | 2.73 [-8.15,13.6] | 0.710 | -0.07 [-0.22,0.07] | 0.844 | 0.01 [-0.02,0.04] | 0.511 | 0.02 [-0.04,0.08] | 0.313 |  |  |
| Q3 vs Q1 | -8.30 [-44.1,27.5] |  | -4.33 [-16.3,7.61] |  | -0.12 [-0.26,0.03] |  | -0.002 [-0.03,0.03] |  | 0.01 [-0.04,0.06] |  |  |  |
| Q4 vs Q1 | 9.88 [-25.7,45.5] |  | 5.05 [-5.40,15.5] |  | 0.01 [-0.13,0.15] |  | 0.02 [-0.03,0.07] |  | 0.06 [-0.05,0.18] |  |  |  |
| **∑PCBs;** Q2 vs Q1 | -23.0 [-59.5,13.5] | 0.364 | -6.03 [-16.6,4.54] | 0.099 | -0.13 [-0.25,-0.01]** | **0.059** | 0.004 [-0.03,0.04] | 0.655 | -0.01 [-0.07,0.05] | 0.937 |  |  |
| Q3 vs Q1 | -3.58 [-45.7,38.6] |  | -9.22 [-20.5,2.11] |  | -0.17 [-0.30,-0.04]** |  | -0.03 [-0.07,0.01] |  | -0.05 [-0.12,0.02] |  |  |  |
| Q4 vs Q1 | -29.2 [-75.4,17.0] |  | -10.5 [-22.1,1.12] |  | -0.17 [-0.33,-0.01]** |  | -0.01 [-0.08,0.06] |  | -0.002 [-0.15,0.15] |  |  |  |
| **∑3MC inducers**; Q2 vs Q1 | -2.52 [-38.8,33.7] | 0.885 | -2.54 [-13.1,8.05] | 0.726 | 0.04 [-0.09,0.16] | 0.124 | -0.0001 [-0.04,0.04] | 0.945 | 0.003 [-0.06,0.07] | 0.773 |  |  |
| Q3 vs Q1 | 5.35 [-34.7,45.4] |  | -3.47 [-14.8,7.85] |  | -0.14 [-0.25,-0.02]** |  | -0.01 [-0.06,0.04] |  | -0.002 [-0.10,0.10] |  |  |  |
| Q4 vs Q1 | -5.58 [-51.9,40.7] |  | -2.35 [-15.2,10.5] |  | -0.08 [-0.23,0.08] |  | -0.0002 [-0.08,0.08] |  | 0.03 [-0.15,0.21] |  |  |  |
| **∑DL-PCBs;** Q2 vs Q1 | -6.16 [-42.6,30.2] | 0.951 | -3.25 [-13.8,7.28] | 0.765 | -0.01 [-0.12,0.10] | 0.145 | -0.01 [-0.04,0.03] | 0.834 | -0.01 [-0.07,0.06] | 0.853 |  |  |
| Q3 vs Q1 | 1.38 [-38.5,41.2] |  | -5.90 [-17.2,5.44] |  | -0.12 [-0.25,0.01] |  | -0.02 [-0.06,0.03] |  | -0.01 [-0.10,0.08] |  |  |  |
| Q4 vs Q1 | -1.07 [-47.1,45.0] |  | -1.71 [-14.2,10.8] |  | -0.09 [-0.24,0.06] |  | -0.01 [-0.08,0.07] |  | 0.02 [-0.15,0.19] |  |  |  |
| **∑NDL-PCBs;** Q2 vs Q1 | -22.2 [-57.9,13.4] | 0.501 | -6.13 [-16.3,4.03] | 0.227 | -0.10 [-0.22,0.02] | 0.120 | 0.001 [-0.03,0.04] | 0.452 | -0.02 [-0.08,0.05] | 0.733 |  |  |
| Q3 vs Q1 | -5.80 [-48.0,36.4] |  | -11.9 [-23.3,-0.48]** |  | -0.16 [-0.29,-0.03]** |  | -0.03 [-0.06,0.01] |  | -0.04 [-0.11,0.02] |  |  |  |
| Q4 vs Q1 | -23.1 [-67.8,21.6] |  | -7.35 [-18.6,3.86] |  | -0.13 [-0.29,0.02] |  | -0.02 [-0.09,0.04] |  | -0.03 [-0.17,0.12] |  |  |  |
| **∑PHB-PCB inducers;** Q2 vs Q1 | -28.5 [-64.8,7.79] | 0.201 | -7.88 [-17.9,2.10] | 0.067 | -0.05 [-0.19,0.09] | 0.208 | 0.01 [-0.03,0.04] | 0.286 | -0.01 [-0.08,0.06] | 0.530 |  |  |
| Q3 vs Q1 | -20.3 [-64.8,24.2] |  | -12.3 [-24.6,-0.11]** |  | -0.11 [-0.24,0.02] |  | -0.03 [-0.07,0.001] |  | -0.06 [-0.12,0.001] |  |  |  |
| Q4 vs Q1 | -38.5 [-85.8,8.70] |  | -11.4 [-22.9,-0.03]** |  | -0.10 [-0.28,0.07] |  | -0.02 [-0.08,0.04] |  | -0.04 [-0.17,0.10] |  |  |  |
| **PBB 153;** Q2 vs Q1 | 48.2 [13.2,83.2]** | 0.223 | 10.5 [0.02,21.0]** | 0.440 | 0.08 [-0.08,0.24] | 0.164 | -0.01 [-0.06,0.04] | 0.969 | -0.002 [-0.10,0.10] | 0.827 |  |  |
| Q3 vs Q1 | 58.6 [17.1,100.1]** |  | 7.78 [-3.58,19.1] |  | 0.04 [-0.14,0.21] |  | -0.02 [-0.07,0.04] |  | -0.02 [-0.13,0.09] |  |  |  |
| Q4 vs Q1 | 33.5 [-5.03,72.0] |  | 7.72 [-3.56,19.0] |  | 0.14 [-0.03,0.31] |  | -0.0001 [-0.04,0.04] |  | 0.01 [-0.06,0.08] |  |  |  |
| **β-HCCH;** Q2 vs Q1 | -53.2 [-94.9,-11.6]** | **0.0001** | -6.81 [-18.7,5.09] | **0.003** | -0.01 [-0.17,0.15] | 0.425 | 0.001 [-0.04,0.04] | 0.358 | 0.004 [-0.08,0.09] | 0.351 |  |  |
| Q3 vs Q1 | -72.5 [-118.5,-26.4]** |  | -14.2 [-28.6,0.23] |  | -0.05 [-0.20,0.10] |  | 0.04 [-0.07,0.14] |  | 0.08 [-0.17,0.33] |  |  |  |
| Q4 vs Q1 | -92.6 [-143.2,-42.1]** |  | -23.3 [-38.7,-7.94]** |  | -0.06 [-0.21,0.11] |  | 0.05 [-0.06,0.15] |  | 0.11 [-0.13,0.35] |  |  |  |
| **HCB;** Q2 vs Q1 | -16.6 [-54.7,21.4] | 0.638 | 4.59 [-5.94,15.1] | 0.498 | -0.04 [-0.19,0.12] | 0.330 | -0.01 [-0.04,0.03] | 0.207 | -0.02 [-0.08,0.04] | 0.358 |  |  |
| Q3 vs Q1 | 6.70 [-34.8,48.2] |  | 12.2 [-1.41,25.8] |  | -0.01 [-0.17,0.15] |  | -0.04 [-0.07,-0.01]** |  | -0.08 [-0.14,-0.02]** |  |  |  |
| Q4 vs Q1 | -29.3 [-76.3,17.6] |  | -1.77 [-18.6,15.1] |  | -0.13 [-0.30,0.03] |  | -0.01 [-0.07,0.06] |  | 0.003 [-0.13,0.14] |  |  |  |
| **Oxychlordane;** Q2 vs Q1 | -18.2 [-53.8,17.4] | 0.408 | -8.47 [-19.1,2.20] | **0.071** | -0.03 [-0.17,0.10] | 0.826 | -0.01 [-0.04,0.02] | 0.417 | -0.004 [-0.05,0.04] | 0.872 |  |  |
| Q3 vs Q1 | -25.1 [-68.0,17.7] |  | -15.4 [-27.9,-2.97]** |  | -0.04 [-0.18,0.10] |  | -0.03 [-0.06,0.01] |  | -0.03 [-0.09,0.03] |  |  |  |
| Q4 vs Q1 | -18.0 [-63.7,27.6] |  | -10.8 [-24.5,2.89] |  | -0.02 [-0.19,0.15] |  | -0.03 [-0.09,0.05] |  | -0.01 [-0.16,0.15] |  |  |  |
| **Trans-nonachlor;** Q2 vs Q1 | -15.3 [-55.7,25.0] | 0.804 | -13.1 [-24.0,-2.25]** | 0.337 | -0.16 [-0.28,-0.03]** | 0.132 | 0.01 [-0.03,0.04] | 0.624 | 0.01 [-0.05,0.07] | 0.985 |  |  |
| Q3 vs Q1 | 0.35 [-40.0,40.7] |  | -7.52 [-19.1,4.09] |  | -0.15 [-0.30,-0.01]** |  | -0.03 [-0.06,0.001] |  | -0.04 [-0.10,0.01] |  |  |  |
| Q4 vs Q1 | -11.7 [-57.0,33.6] |  | -8.97 [-21.9,3.95] |  | -0.14 [-0.30,0.03] |  | -0.01 [-0.08,0.07] |  | 0.02 [-0.15,0.19] |  |  |  |
| **p,p’-DDE;** Q2 vs Q1 | -26.7 [-67.8,14.3] | 0.131 | -2.64 [-15.4,10.1] | 0.132 | -0.05 [-0.21,0.11] | **0.016** | -0.01 [-0.04,0.02] | 0.636 | -0.02 [-0.07,0.03] | 0.446 |  |  |
| Q3 vs Q1 | -33.9 [-77.1,9.32] |  | -4.74 [-16.6,7.16] |  | -0.13 [-0.28,0.01] |  | -0.02 [-0.06,0.02] |  | -0.03 [-0.11,0.04] |  |  |  |
| Q4 vs Q1 | -37.9 [-85.4,9.58] |  | -10.7 [-25.3,4.00] |  | -0.18 [-0.34,-0.02]** |  | 0.02 [-0.07,0.11] |  | 0.08 [-0.13,0.29] |  |  |  |

^a^ Multivariable models adjusted for age, body mass index, waist-to-hip ratio, acculturation score Multiethnic Study of atherosclerosis, study sites, Hispanic/Latino background, educational attainment, estimated glomerular filtration rate, smoking status, alcohol consumption, physical activity levels, and alternative healthy eating index 2010

^b^ POPs concentrations were evaluated in quartiles as follows - Q4: quartile 4; Q3: quartile 3; Q2: quartile 2; Q1: quartile 1

^c^ Ln transformed SHBG, LH and FSH were modeled in men. Estimates for associations of POPs serum concentration with Ln transformed hormones were back transformed $[100 \left( e^{\beta POPS}-1 \right)\%]$ and can be interpreted as a quartile increase in POPs concentration is associated with relative change in median or geometric mean of hormone corresponding to $\beta\%$

∑PBDEs — PBDE 100, 153, 154, 17, 183, 209, 28, 47, 85, 99

∑PCBs — PCB 105, 114, 118, 138-158, 156, 153, 156, 157, 167, 170, 178, 180, 183, 187, 189, 194, 196-203, 199, 206, 209, 28, 66, 74, 99

∑3-methylcholanthrene (3MC) inducers: CYP IA inducers/substrates — PCB 66, 74, 105, 118, 156, 167

∑Dioxin-like PCBs — PCB 105, 114, 118, 156, 157, 167, 189

∑Non-Dioxin-like PCBs — PCB 28, 66, 74, 99, 138158, 146, 153, 170, 178, 180, 183, 187, 194, 196-203, 199, 206, 209

∑Phenobarbital-type (PB) inducers: CYP IIB inducers – PCB 99, 153, 180, 183, 187, 194, 196–203, 199, 206

** p<0.05; * p<0.10

**Table S12. Multivariable logistic regression models evaluating associations^a^ of POPs serum concentrations with sex-related hormones, Hispanic/Latino postmenopausal women (N=716)**

| **POPs concentrations (ng/g Lipid)** | **Low SHBG**^c^ | **Low DHEAS**^c^ |
| --- | --- | --- |
|  | **OR (95% CI)** | **OR (95% CI)** |
| **∑PBDEs** | 1.17 [0.73,1.89] | 0.85 [0.21,3.45] |
| **∑PCBs** | 0.38 [0.14,1.02] | 1.47 [0.52,4.13] |
| **∑3MC-PCB inducers** | 0.49 [0.16,1.49] | 1.19 [0.45,3.15] |
| **∑Dioxin-like PCBs** | 0.45 [0.15,1.40] | 1.63 [0.64,4.15] |
| **∑Non-Dioxin-like PCBs** | 0.42 [0.17,1.04] | 1.57 [0.57,4.35] |
| **∑PHB-PCB inducers** | 0.42 [0.16,1.12] | 1.30 [0.42,4.03] |
| **PBB 153** | 0.65 [0.29,1.43] | 0.43 [0.10,1.92] |
| **β-HCCH** | 0.55 [0.24,1.28] | 0.61 [0.24,1.52] |
| **HCB** | 0.55 [0.22,1.34] | 0.45 [0.17,1.20] |
| **Oxychlordane** | 0.33 [0.14,0.74]** | 0.49 [0.17,1.44] |
| **Trans-nonachlor** | 0.61 [0.32,1.14] | 1.19 [0.46,3.10] |
| **p,p’-DDE** | 0.93 [0.40,2.18] | 0.33 [0.13,0.81]** |

^a^ Multivariable models adjusted for age, body mass index, waist-to-hip ratio, acculturation score Multiethnic Study of atherosclerosis, study sites, Hispanic/Latino background, educational attainment, estimated glomerular filtration rate, smoking status, alcohol consumption, physical activity levels, alternative healthy eating index 2010 and number of live births (women only)

^b^ POPs exposures were assessed from POPs serum concentration quartiles and interpreted as per quartile increase in concentration

^c^ Low SHBG was categorized as less than 20 nmol/L (2% of postmenopausal women) and low DHEAS as less than 0.26 umol/L (2% of postmenopausal women)

∑PBDEs — PBDE 100, 153, 154, 17, 183, 209, 28, 47, 85, 99

∑PCBs — PCB 105, 114, 118, 138-158, 156, 153, 156, 157, 167, 170, 178, 180, 183, 187, 189, 194, 196-203, 199, 206, 209, 28, 66, 74, 99

∑3-methylcholanthrene (3MC) inducers: CYP IA inducers/substrates — PCB 66, 74, 105, 118, 156, 167

∑Dioxin-like PCBs — PCB 105, 114, 118, 156, 157, 167, 189

∑Non-Dioxin-like PCBs — PCB 28, 66, 74, 99, 138158, 146, 153, 170, 178, 180, 183, 187, 194, 196-203, 199, 206, 209

∑Phenobarbital-type (PB) inducers: CYP IIB inducers – PCB 99, 153, 180, 183, 187, 194, 196–203, 199, 206

** p<0.05; *p<0.10

**Table S13. Multivariable logistic regression models evaluating associations^a^ of POPs serum concentrations with sex-related hormones, Hispanic/Latino men (N=1,073)**

| **POPs concentrations (ng/g Lipid)** | **High LH**^c^ | **High FSH**^c^ | **High E2**^c^ | **Low T**^c^ |
| --- | --- | --- | --- | --- |
|  | **OR (95% CI)** | **OR (95% CI)** | **OR (95% CI)** | **OR (95% CI)** |
| **∑PBDEs** | 0.94 [0.75,1.18] | 1.00 [0.78,1.30] | 1.12 [0.74,1.69] | 1.28 [0.84,1.94] |
| **∑PCBs** | 0.86 [0.65,1.14] | 1.03 [0.73,1.44] | 0.51 [0.35,0.74]** | 1.09 [0.65,1.84] |
| **∑3MC-PCB inducers** | 0.91 [0.71,1.17] | 0.90 [0.65,1.26] | 0.69 [0.48,0.99]** | 0.77 [0.44,1.36] |
| **∑Dioxin-like PCBs** | 0.93 [0.72,1.19] | 0.90 [0.65,1.26] | 0.62 [0.43,0.90]** | 0.73 [0.43,1.26] |
| **∑Non-Dioxin-like PCBs** | 0.83 [0.63,1.08] | 0.92 [0.68,1.26] | 0.49 [0.34,0.71]** | 1.07 [0.65,1.77] |
| **∑PHB-PCB inducers** | 0.81 [0.61,1.06] | 0.90 [0.65,1.24] | 0.42 [0.28,0.65]** | 1.23 [0.78,1.94] |
| **PBB 153** | 0.86 [0.67,1.10] | 0.77 [0.56,1.05] | 0.89 [0.52,1.53] | 1.22 [0.74,2.03] |
| **β-HCCH** | 0.87 [0.63,1.20] | 0.95 [0.59,1.53] | 1.10 [0.72,1.70] | 0.90 [0.54,1.51] |
| **HCB** | 1.02 [0.77,1.35] | 0.89 [0.57,1.39] | 1.02 [0.60,1.72] | 0.55 [0.34,0.88]** |
| **Oxychlordane** | 0.89 [0.69,1.15] | 0.9 [0.61,1.32] | 1.10 [0.76,1.60] | 0.84 [0.52,1.37] |
| **Trans-nonachlor** | 0.88 [0.69,1.14] | 0.99 [0.70,1.41] | 1.00 [0.70,1.44] | 0.81 [0.50,1.32] |
| **p,p’-DDE** | 0.82 [0.62,1.09] | 1.02 [0.63,1.67] | 1.24 [0.82,1.87] | 1.08 [0.59,1.97] |

^a^ Multivariable models adjusted for age, body mass index, waist-to-hip ratio, acculturation score Multiethnic Study of atherosclerosis, study sites, Hispanic/Latino background, educational attainment, estimated glomerular filtration rate, smoking status, alcohol consumption, physical activity levels, and alternative healthy eating index 2010

^b^ POPs exposures were assessed from POPs serum concentration quartiles and interpreted as per quartile increase in concentration

^c^ High LH was categorized as greater than 8.6 mlU/mL (20% of men), high FSH as greater than 12.4 mlU/mL (10% of men), high E2 as greater than 159 pmol/L (5% of men), and low T as less than 193 ng/dL (4% of men)

∑PBDEs — PBDE 100, 153, 154, 17, 183, 209, 28, 47, 85, 99

∑PCBs — PCB 105, 114, 118, 138-158, 156, 153, 156, 157, 167, 170, 178, 180, 183, 187, 189, 194, 196-203, 199, 206, 209, 28, 66, 74, 99

∑3-methylcholanthrene (3MC) inducers: CYP IA inducers/substrates — PCB 66, 74, 105, 118, 156, 167

∑Dioxin-like PCBs — PCB 105, 114, 118, 156, 157, 167, 189

∑Non-Dioxin-like PCBs — PCB 28, 66, 74, 99, 138158, 146, 153, 170, 178, 180, 183, 187, 194, 196-203, 199, 206, 209

∑Phenobarbital-type (PB) inducers: CYP IIB inducers – PCB 99, 153, 180, 183, 187, 194, 196–203, 199, 206

** p<0.05; *p<0.10
